# Heat-responsive microRNAs participate in regulating the pollen fertility stability of CMS-D2 restorer line under high-temperature stress

**DOI:** 10.1186/s40659-023-00465-y

**Published:** 2023-11-09

**Authors:** Meng Zhang, Xuexian Zhang, Ruijie Wang, Rong Zang, Liping Guo, Tingxiang Qi, Huini Tang, Liangliang Chen, Hailin Wang, Xiuqin Qiao, Jianyong Wu, Chaozhu Xing

**Affiliations:** National Key Laboratory of Cotton Bio-breeding and Integrated Utilization, Institute of Cotton Research of Chinese Academy of Agricultural Sciences, Key Laboratory for Cotton Genetic Improvement, Ministry of Agriculture and Rural Affairs, 38 Huanghe Dadao, Anyang, 455000 Henan China

**Keywords:** CMS-D2 restorer line, Pollen fertility stability, MiRNA cluster, High-temperature stress, Degradome, Plant hormone signal transduction

## Abstract

Anther development and pollen fertility of cytoplasmic male sterility (CMS) conditioned by *Gossypium harknessii* cytoplasm (CMS-D2) restorer lines are susceptible to continuous high-temperature (HT) stress in summer, which seriously hinders the large-scale application of "three-line" hybrids in production. Here, integrated small RNA, transcriptome, degradome, and hormone profiling was performed to explore the roles of microRNAs (miRNAs) in regulating fertility stability in mature pollens of isonuclear alloplasmic near-isogenic restorer lines NH and SH under HT stress at two environments. A total of 211 known and 248 novel miRNAs were identified, of which 159 were differentially expressed miRNAs (DEMs). Additionally, 45 DEMs in 39 miRNA clusters (PmCs) were also identified, and most highly expressed miRNAs were significantly induced in SH under extreme HT, especially four MIR482 and six MIR6300 family miRNAs. PmC28 was located in the fine-mapped interval of the *Rf*_*1*_ gene and contained two DEMs, gra-miR482_L-2R + 2 and gma-miR2118a-3p_R + 1_1ss18TG. Transcriptome sequencing identified 6281 differentially expressed genes, of which heat shock protein (HSP)-related genes, such as *HSP70*, *HSP22*, *HSP18.5-C*, *HSP18.2* and *HSP17.3-B*, presented significantly reduced expression levels in SH under HT stress. Through integrating multi-omics data, we constructed a comprehensive molecular network of miRNA–mRNA–gene-KEGG containing 35 pairs of miRNA/target genes involved in regulating the pollen development in response to HT, among which the mtr-miR167a_R + 1, tcc-miR167c and ghr-miR390a, tcc-miR396c_L-1 and ghr-MIR169b-p3_1ss6AG regulated the pollen fertility by influencing *ARF8* responsible for the auxin signal transduction, ascorbate and aldarate metabolism, and the sugar and lipid metabolism and transport pathways, respectively. Further combination with hormone analysis revealed that HT-induced jasmonic acid signaling could activate the expression of downstream auxin synthesis-related genes and cause excessive auxin accumulation, followed by a cascade of auxin signal transduction, ultimately resulting in pollen abortion. The results provide a new understanding of how heat-responsive miRNAs regulate the stability of fertility restoration for CMS-D2 cotton under heat stress.

**Supplementary Information:**

The online version contains supplementary material available at 10.1186/s40659-023-00465-y.

## Background

Cotton (*Gossypium hirsutum* L.), one of the most important economic crops, is widely grown worldwide [1]. Utilization of heterosis can significantly increase cotton yield, improve fiber quality and enhance stress resistance [2, 3]. As an ideal pollination control system, the use of CMS for hybrid seed production can not only save the tedious steps of manual emasculation and reduce labor costs but also can effectively improve the purity of hybrids [4]. In cotton, CMS-D2 is the main source of sterile cytoplasm for cotton "three-line" hybrids currently grown in China [5–7]. However, the anther and/or pollen development of CMS-D2 restorer lines and hybrids is easily affected by continuous HT stress in summer [8], which seriously hampers the large-scale application of "three-line" hybrids in China.

Male reproductive development in flowering plants, especially at the young microspore stage has been testified to be extremely vulnerable to HT stress [9–11]. Generally, anther indehiscence, shortened filaments, microspore abortion, rudimentary pollen tube formation and premature or delayed tapetum degradation all occur when encountering sustained heat stress [8, 12, 13]. Basic thermo-tolerance responses in plants have been shown to involve a range of biochemical molecular changes in nearly all biological processes, such as heat shock factors (HSFs) and HSPs, calcium, reactive oxygen species (ROS), nitric oxide (NO), unfolded protein response (UPR) and cytoplasmic protein response (CPR) signaling pathways [14]. Besides, major endogenous phytohormones including jasmonic acid (JA) [15–17], auxin [18–20] and gibberellin acid (GA) [21] were also reported to be involved in HT response during anther development. In barley and model plant *Arabidopsis thaliana*, male sterility was caused by HT resulting from suppressing endogenous auxin biosynthesis and exogenous auxin application can completely rescue male fertility under HT stress [18]. Conversely, it was demonstrated that *PHYTOCHROME-INTERACTING FACTOR (PIF)* genes through the sugar signaling pathway can stimulate indole-3-acetic acid (IAA) biosynthesis during HT stress which eventually caused male sterility in cotton [19]. Another study found that loss of *Arabidopsis* AUXIN RESPONSE FACTOR 6 (*ARF6*) and *ARF8* disrupts JA production and hence causes delayed or non-dehiscence, and reduced filament and petal elongation as seen in the JA-deficient mutant *dad1* [22], which can also be restored by exogenous JA application [23]. Therefore, auxin and JA biosynthesis and subsequent signal transduction pathways can be considered to play critical roles in regulating anther development, but the molecular mechanism of how these two work together in cotton under HT stress is still unclear.

MiRNAs are an extensive class of endogenous short non-coding RNAs, typically with approximately 21–24 nucleotides (nt), that can regulate gene expression post-transcriptionally by cutting mRNA or inhibiting translation mainly through sequence complementarity [24], and then participate in plant growth and development [25] and response to abiotic stresses [26]. Recently, a large number of miRNAs have been identified and/or confirmed to be involved in the regulation of male fertility in response to HT stress through small RNA sequencing techniques in various plant species, including rice [27], barley [28], soybean [29] tomato [30], and cotton [20, 31]. However, only a few miRNAs have been well functionally validated in plant responses to heat stress so far. In *Arabidopsis*, miR398 was found to be rapidly induced by heat stress and make plants more sensitive to HT by suppressing the expression levels of genes encoding copper superoxide dismutase, mainly as an important class of important ROS scavengers [32]. Another important miRNA, miR156 can regulate tolerance to recurring heat stress through SPL transcription factor genes and has been presented to be highly induced by HT in *Arabidopsis* [33]. In contrast, all members of the miR156 family were inhibited by HT in soybean flower buds, and overexpression of gma-miR156b in *Arabidopsis* resulted in male sterility under HT stress [29]. Furthermore, some heat-responsive miRNAs in plants, including miR160, miR167 and miR393, have been reported to be involved in auxin signal transduction pathway, and their targets are mainly auxin receptors or signal-responsive genes, such as *TRANSPORT INHIBITOR RESISTANT 1* (*TIR1*), *AUXIN SIGNALING F-BOX* (*AFB*), and *AUXIN RESPONSE FACTORS* (*ARFs*) [20, 34]. Although many miRNAs have been found in plant response to HT during anther development, the regulatory roles of miRNAs associated with male fertility stability in cotton, especially CMS-D2 restorer line under HT stress, remains largely obscure.

Our previous research has revealed that DNA methylation is involved in the anther fertility of restorer line with sterile cytoplasm under HT stress [8]. In this study, a comparative analysis of the integrated small RNA, transcriptome, degradome, and hormone profiling was implemented to explore the roles of miRNAs in regulating pollen fertility stability in mature pollens of isonuclear alloplasmic near-isogenic restorer lines NH and SH, which exhibited significantly different male fertility phenotypes in response to HT. These analyses identified and characterized differentially expressed miRNAs (DEMs), miRNA clusters and their targets, and further, constructed a comprehensive molecular network of miRNA–mRNA–gene-KEGG containing 35 pairs of miRNA/target genes involved in the regulation of pollen development under heat stress. Further combination with hormone data, we propose that HT-induced JA signaling could trigger the expression of downstream auxin synthesis-related genes and cause excessive auxin accumulation, followed by a cascade of auxin signal transduction, ultimately leading to pollen abortion. The results of this study provide valuable information for further elucidating the regulatory mechanisms of the negative effects of sterile cytoplasm on the pollen development of CMS-D2 restorer lines or hybrids, and the obtained epigenetic resources here will help accelerate the breeding of heat-tolerant cotton restorer lines by improving the male fertility stability through epigenetic engineering methods, so as to increase the yield of "three-line" hybrids to cope with the current and future global warming situation.

## Results

### Phenotypic comparison of male fertility in cotton restorer lines with different cytoplasm under HT stress

**Fig. 1:**
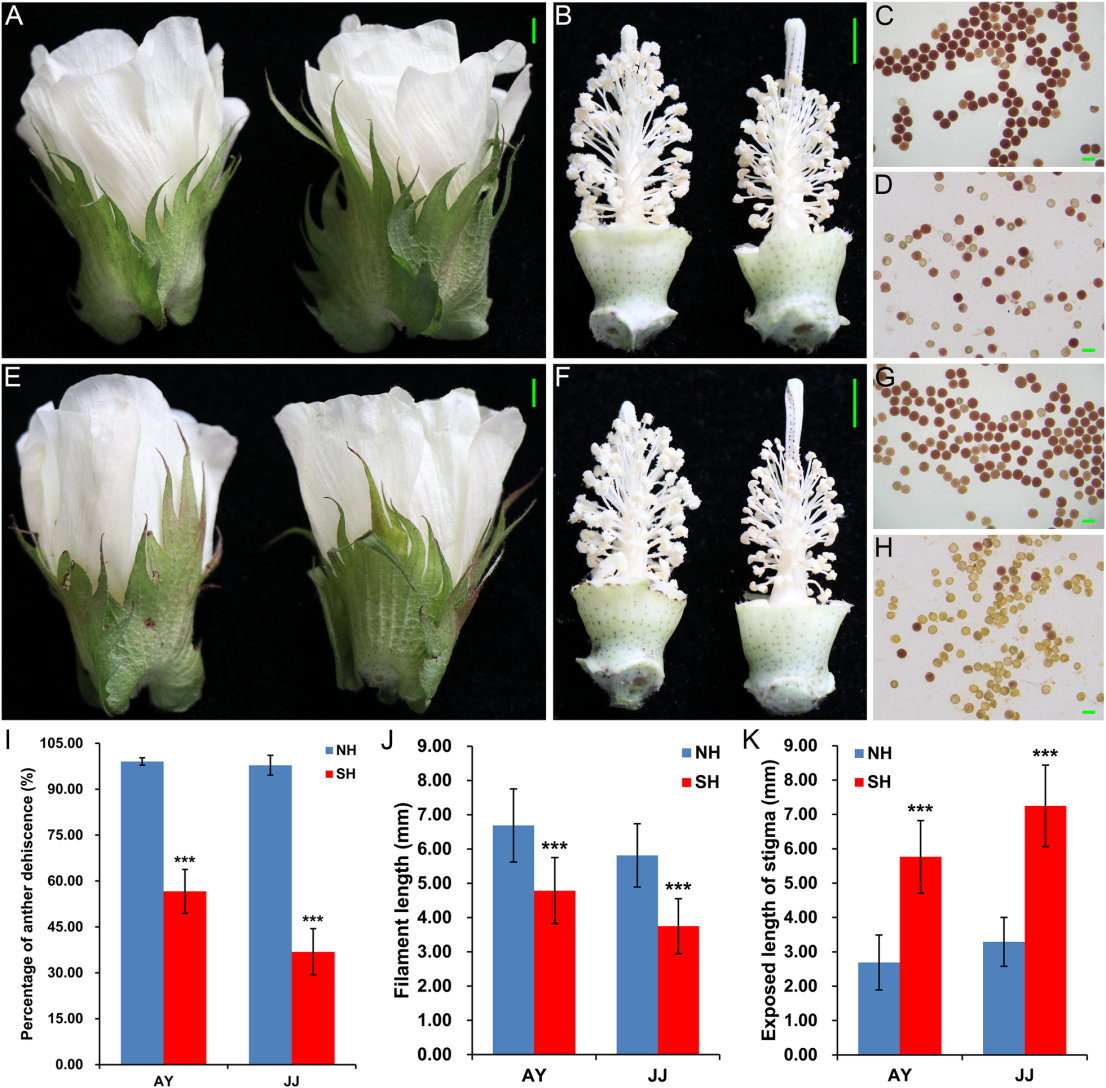
Comparison of the field performance of flowers and anthers from NH and its isonuclear alloplasmic near-isogenic line SH under mild HT (AY) and extreme HT (JJ) stress. **A** A representative intact flower from NH (left) and SH (right) under mild HT in AY. **B** Anthers from NH (left) and SH (right), showing normal pollen release only from NH under mild HT in AY. **C**, **D** Pollen grains from NH (**C**) and SH (**D**) plants under mild HT stress and stained with Benzidine-α-Naphthol in AY. **E** A representative intact flower from NH (left) and SH (right) under extreme HT in JJ. **F** Anthers from NH (left) and SH (right), showing normal pollen release only from NH under extreme HT in JJ. **G**, **H** Pollen grains from NH (**G**) and SH (**H**) plants under extreme HT stress and stained with Benzidine-α-Naphthol in JJ. More sterile pollen grains from SH were observed under HT stress, especially in JJ spot. Fertile pollen is stained red, part of the vitality shows reddish, and sterile pollen is colorless. Scale bars = 5 mm in **A**, **B**, **E**, **F** and 100 µm in **C**, **D**, **G**, **H**. **I**–**K** Graphical representations of the percentage of anther dehiscence (**I**), filament length (**J**), and exposed length of stigma (**K**) of NH and SH under mild and extreme HT stress in AY and JJ spots, respectively. *AY* Anyang, *JJ* Jiujiang

Our previous studies have found that the male fertility of cotton restorer line with sterile cytoplasm is susceptible to continuous external HT stress [8]. To further explore the potential mechanism of pollen fertility instability under mild and extreme HT stress, two isonuclear alloplasmic near-isogenic cotton restorer lines with obvious differences in anther phenotypes under external HT stress were used, namely, NH (HT-tolerant) and SH (HT-sensitive) (Fig. 1). At the two ecological spots, AY and JJ, there was generally no obvious difference in the external morphology and size of the intact flowers of NH and SH under mild or extreme HT stress (Fig. 1A, E). However, the anther and pollen fertility of the restorer line SH with sterile cytoplasm was significantly lower than that of normal upland cotton cytoplasm NH, especially under extreme HT stress in JJ spot where the summer temperature is higher (Fig. 1B-D, F–H). In addition, whether in the AY with a mild HT or the JJ with an extreme HT, it was observed that compared with NH, SH showed a significant decrease in the normal anther dehiscence ratio and filament length, and a significant increase in the exposed length of the stigma (Fig. 1I–K). This indicates that the male fertility of the sterile cytoplasmic restorer line SH was significantly affected under HT stress, which is finally manifested in the non-cracking of the anthers and the significantly reduced pollen fertility, whereas the NH performed normally (Fig. 1).

### Global small RNA sequencing data analysis in cotton pollen grains under HT stress

**Fig. 2:**
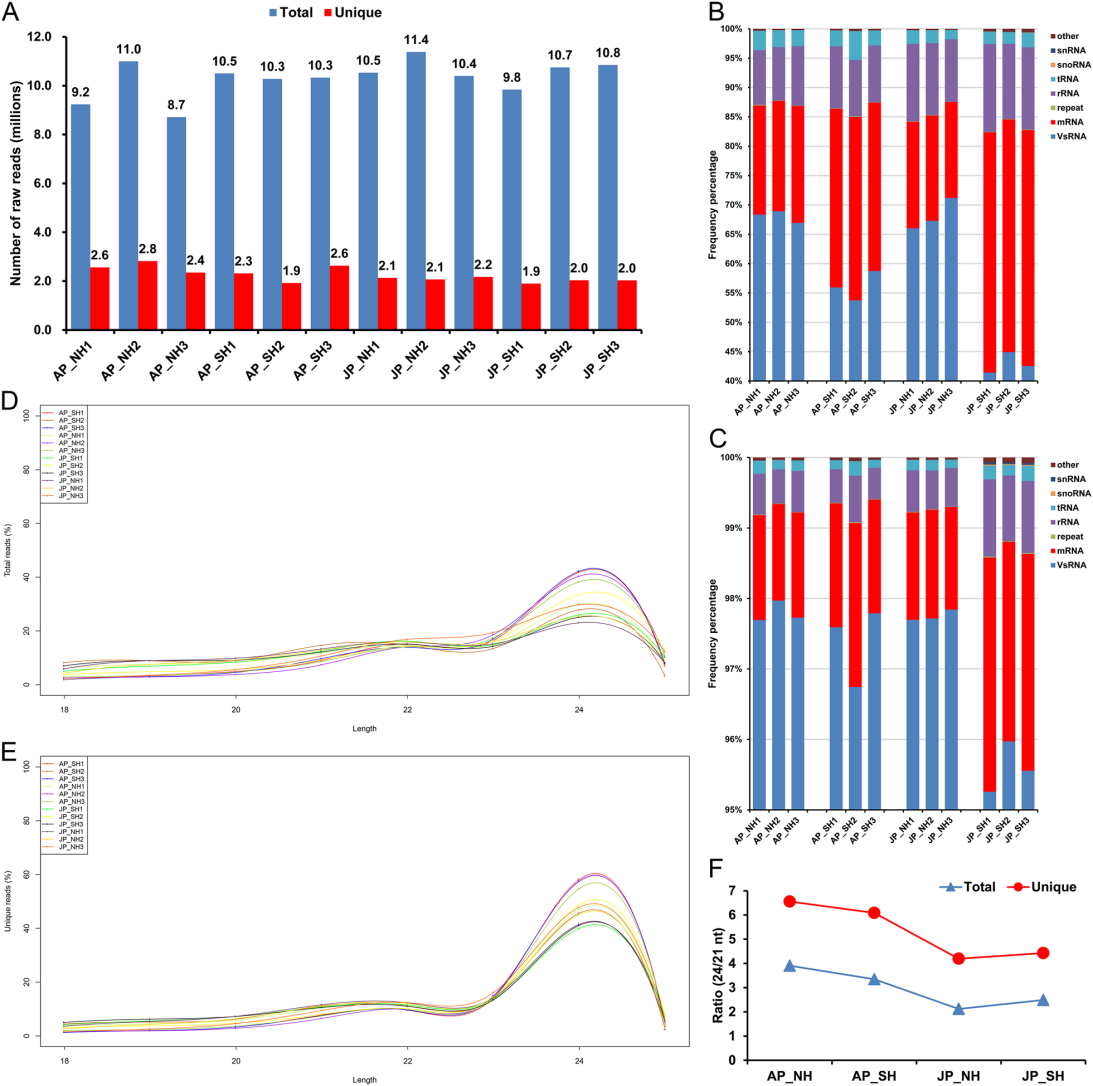
Overview of small RNA sequencing data and length distribution. **A** Statistics of total and unique sRNA reads in 12 sequencing libraries. **B**, **C**) Annotation distribution of the total **B** and unique **C** reads in each sample. other, other Rfam RNA; snRNA, small nuclear RNA; snoRNA, small nucleolar RNA; tRNA, transfer RNA; rRNA, ribosomal RNA; repeat, repeat associate RNA; mRNA, messenger RNA; VsRNA, valid small RNA. **D**, **E** Length distribution of 18–25 nt small RNAs identified in total (**D**) and unique € reads. The X-axis indicates sRNAs of different lengths, while the Y-axis represents the percentage of sRNAs at a certain length. **F** Ratio of 24/21 nt sRNAs. The ratio of NH and SH at the JJ spot is significantly lower than that of AY. Three biological replicates are merged into the mean value. AY, Anyang; JJ, Jiujiang. AP_NH, NH under mild HT stress; AP_SH, SH under mild HT stress; JP_NH, NH under extreme HT stress; JP_SH, SH under extreme HT stress

To explore whether miRNAs are involved in male fertility instability under HT in CMS system cotton, mature pollen grains from NH and SH were collected separately with three biological repeats under mild and extreme HT stress, respectively, and used for small RNA (sRNA) library construction and sequencing to identify changes in miRNA abundance in response to HT. Pearson correlation analysis showed that the correlation coefficients among the three replicates of each sample exceeded 0.95, indicating that the sequencing results were relatively accurate and reliable (Additional file 1: Fig. S1). More than 120 million total raw reads were obtained from the 12 small RNA sequencing (sRNA-seq) libraries, and the average total and unique reads for each sample were approximately 10 and two million, respectively (Fig. 2A, Additional file 2: Table S1). After removing 3′adapter sequences, poly-A tags, small tags less than 18-nt and poor-quality reads from raw data, the clean reads obtained were filtered using mRNA, Rfam and Repbase databases, and the number and percentage of different sRNA reads were classified and counted (Figs. 2, Additional file 1: Figs. S2, S3, Additional file 2: Tables S1, S2). The proportion of valid small RNA (VsRNA) reads in SH was significantly less than that of NH, especially in JJ spot with extreme HT (Fig. 2B, C).

Afterward, sRNAs within a certain length range (18–25 nucleotide (nt)) in total and unique reads were selected and counted (Fig. 2D, E, Additional file 2: Table S3). All samples showed the highest abundance at 24-nt, and sRNAs of 21–24 nt in length accounted for more than 66% of all reads in each library, representing the majority of small RNAs. Additionally, no significant difference was found in the abundance of 21 and 24 nt sRNAs between NH and SH under HT (Fig. 2D, E). Considering 24-nt, small interfering RNA (siRNA) is a class of double-stranded small RNA molecules associated with RNA-directed DNA methylation (RdDM) process [35]; the changes in the ratio of 24/21 nt might reflect changes in DNA methylation levels during pollen development. Hence, the ratios of 24/21 nt sRNAs in each sample were compared. The results showed that NH had the highest ratios (3.91 for total and 6.55 for unique reads) and the lowest ratios (2.12 for total and 4.19 for unique reads) under mild and extreme HT, respectively, and the ratios of NH and SH at JJ ecological spot were significantly lower than that of AY (Fig. 2F), so it is speculated that extensive DNA demethylation occurred during pollen development under HT stress [8].

### Systematic identification of miRNAs in pollen grains of cotton restorer lines

**Fig. 3:**
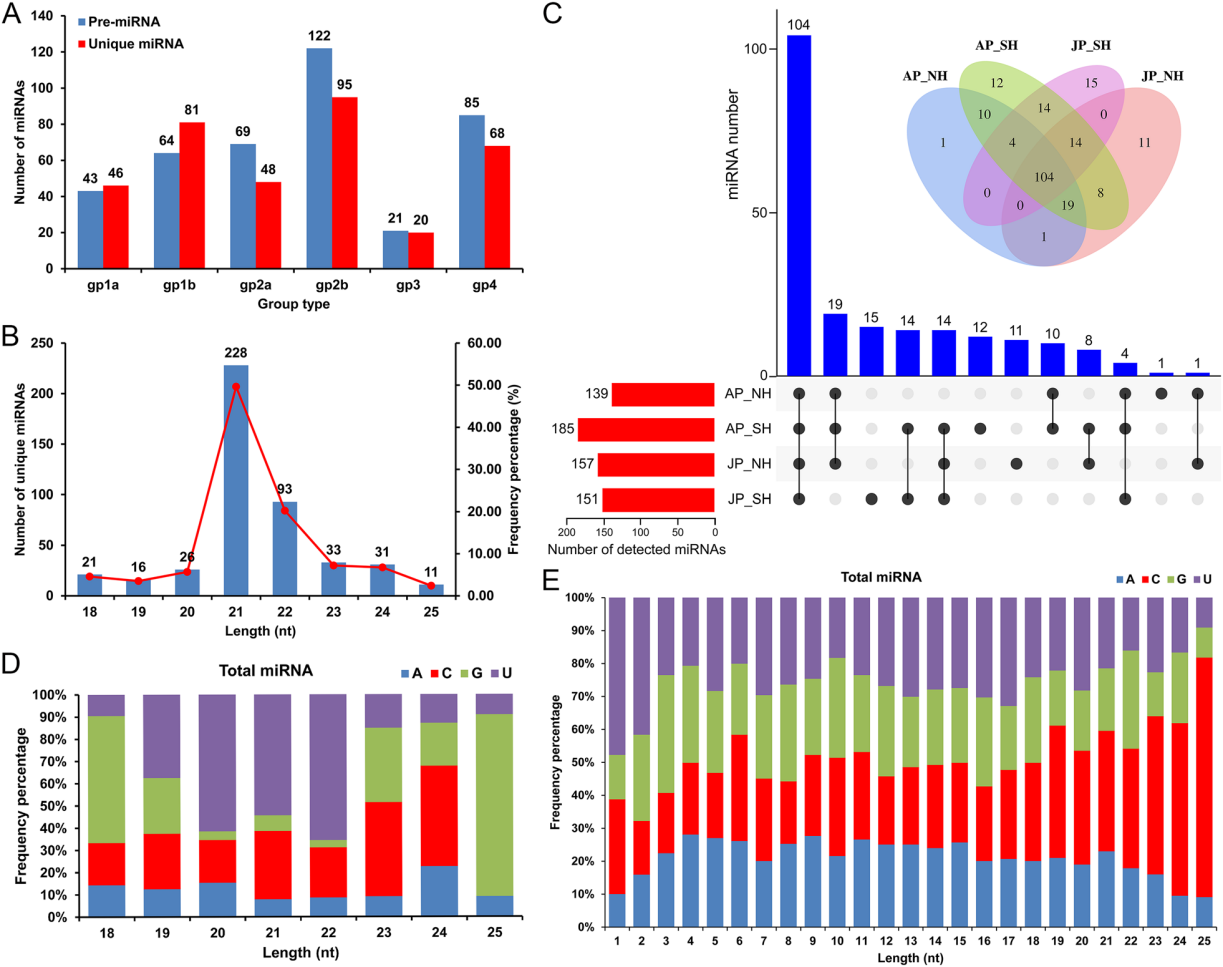
Statistics of identified miRNAs and analysis of their base biases in the sequencing libraries. **A** Number of pre-miRNAs and unique miRNAs in different categories. **B** Length distribution of all identified unique miRNAs. **C** UpSet Venn diagram showing the number of shared and specific miRNAs in different samples. AP_NH, NH under mild HT stress; AP_SH, SH under mild HT stress; JP_NH, NH under extreme HT stress; JP_SH, SH under extreme HT stress. **D** The first base preference of mature miRNAs. The X-axis displays the length classification of the miRNAs, and the Y-axis represents the proportion of mature miRNAs with a certain base type as the first base. **E** Base preference in different positions of mature miRNAs. The X-axis displays the different base positions of the miRNAs, and the Y-axis denotes the proportion of bases at a certain position of mature miRNAs

A total of 404 pre-miRNAs and 459 (358 unique) miRNAs were identified in all samples, including 211 known and 248 newly identified miRNAs (Fig. 3A, Additional file 2: Tables S4, S5). Among the five different categories, the number of pre-miRNAs and unique miRNAs in 'gp2b' was the largest, and 'gp3' was the least, especially in SH (Fig. 3A, Additional file 1: Fig. S4A, B, Additional file 2: Table S5). Almost half of the identified miRNAs were 21-nt in length (228), and 22-nt was the second (93), accounting for about 20.26% (Fig. 3B, Additional file 2: Table S6). Moreover, 104 shared miRNAs were found in all samples, and specific miRNAs accounted for only a small portion (Fig. 3C). Subsequently, the detected miRNAs were analyzed for family and conservation. The results showed that the MIR166 family had the most miRNA members (19), followed by MIR156 and MIR482 with 14 and 13 miRNA members, respectively, and miRNAs were highly conserved among different species (Additional file 1: Fig. S5, Additional file 2: Tables S7–S9).

For all identified miRNAs, the first base preference of the shortest 18-nt (57.14%) and longest 25-nt (81.82%) mature miRNAs was mainly ‘G’; mature 19–22 nt miRNAs mostly started with ‘U’ as the first base (37.50–65.59%), while 23 and 24 nt miRNAs mostly started with ‘C’ (42.42 and 45.16%, respectively) (Fig. 3D, Additional file 2: Table S10). The mature miRNAs of the 'gp1_3' categories were found to have the same first base preference as the above, but the first base of the 'gp4' miRNAs was mainly 'U' or 'C' (Additional file 1: Fig. S4C, D, Additional file 2: Tables S4, S10). Furthermore, the base preference of mature miRNAs at different positions was also counted. Clearly, the 5'start and 3'end bases were mainly 'U' and 'C', respectively, whereas there was no obvious preference for the base distribution in most of the middle positions (Fig. 3E, Additional file 1: Fig. S4E, F, Additional file 2: Tables S4, S11).

### Expression changes of miRNAs in pollen grains in response to HT stress

**Fig. 4:**
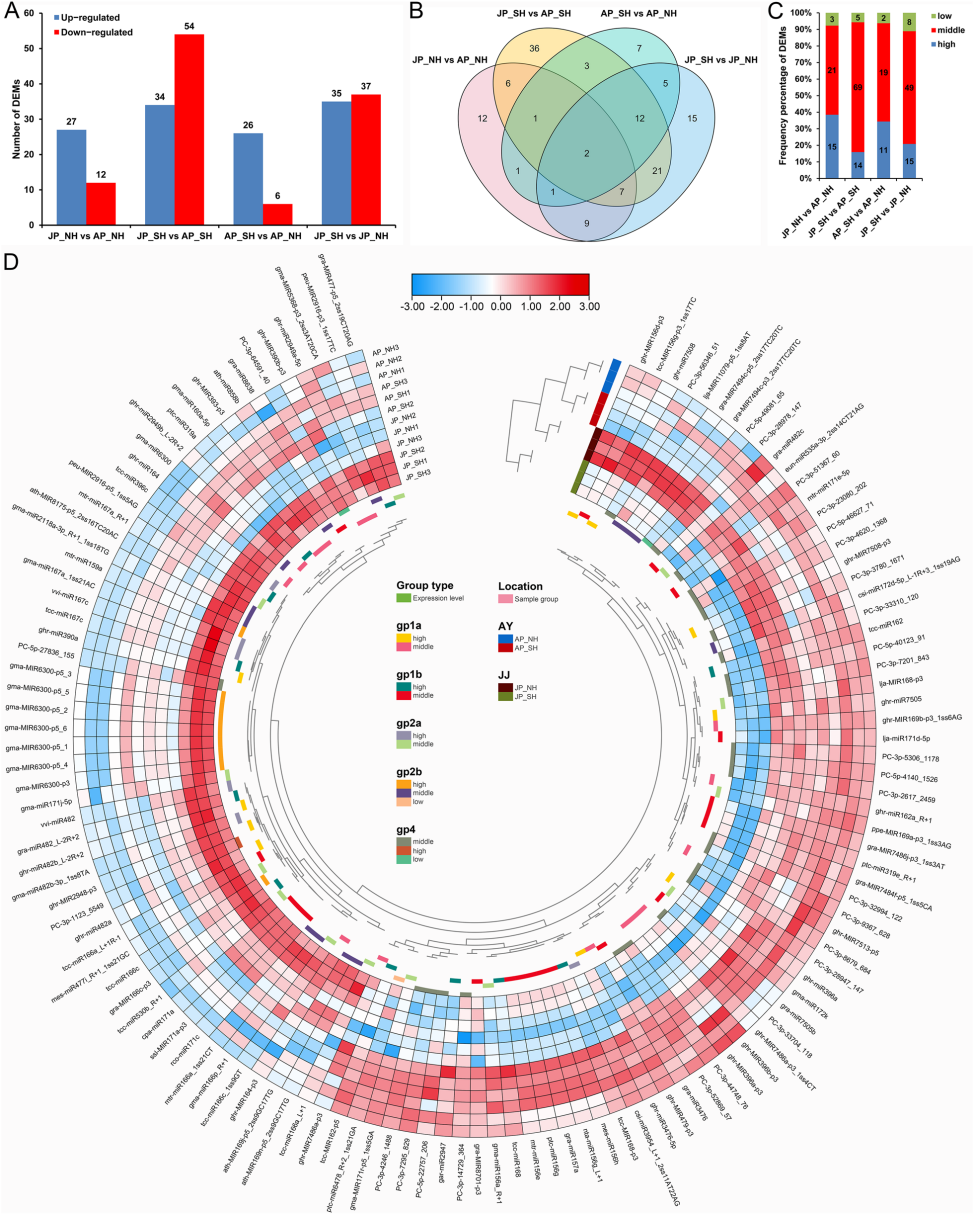
Identification of differentially expressed miRNAs in NH and SH under mild and extreme HT stress. **A** Number of DEMs that were up- or down-regulated under mild and extreme HT stress. **B** Venn diagram showing the number of unique and shared DEMs. **C** Frequency percentage of DEMs with different expression levels. **D** Hierarchical cluster analysis of the identified 126 DEMs using a bending heat map drawn by TBtools software. The relative expression levels in the figure are the normalized expression data displayed with log_10_ (norm + 1) value. AP_NH, NH under mild HT stress; AP_SH, SH under mild HT stress; JP_NH, NH under extreme HT stress; JP_SH, SH under extreme HT stress

To determine the miRNAs that were differentially expressed between NH and SH with different cytoplasm under HT stress, four comparisons were performed and among all 459 expressed miRNAs (200 located in At and 225 in Dt sub-genome), totally 159 differentially expressed miRNAs (DEMs; 126 non-redundant) were identified (Fig. 4, Additional file 2: Table S12), including 74 and 78 that were distributed across the 13 chromosomes of At and Dt sub-genomes of upland cotton, respectively, and seven DEMs on four different scaffolds (Additional file 1: Fig. S6). Remarkably, Chir_D05 (with restorer gene *Rf*_*1*_; eight DEMs out of 26 miRNAs) and its homologous Chir_A05 (10 DEMs out of 27 miRNAs) carried relatively more DEMs than the other chromosomes, which was consistent with our previous sequencing results [8, 36]. Among them, a total of 27 and 12 miRNAs were up- and down-regulated, respectively, in JP_NH versus AP_NH, whereas 34 up- and 54 down-regulated miRNAs were identified in JP_SH versus AP_SH. Under mild HT stress, only 26 up- and six down-regulated miRNAs were identified for the comparison of AP_SH versus AP_NH; comparatively, a total of 72 miRNAs were differentially expressed (35 up- and 37 down-regulated) between NH and SH under extreme HT stress (Fig. 4A). The Venn diagram displayed that the combinations AP_SH versus AP_NH and JP_SH versus JP_NH had only 20 DEMs in common. However, the DEMs only under extreme HT stress (52) accounted for 61.90% of the total DEMs (84) of these two combinations. This indicated that most DEMs between NH and SH had differential expression changes in response to HT (Fig. 4B). The expression levels of more than half of DEMs in all four comparison combinations were moderately expressed, especially under extreme HT stress (Fig. 4C). Hierarchical clustering analysis of genome-wide differential expression levels in all 12 samples is shown in Fig. 4D with a bending heat map. Apparently, most miRNAs between NH and SH were found to have a wide range of expression levels in response to HT stress.

### Whole-genome identification and expression level analysis of miRNA clusters

**Fig. 5:**
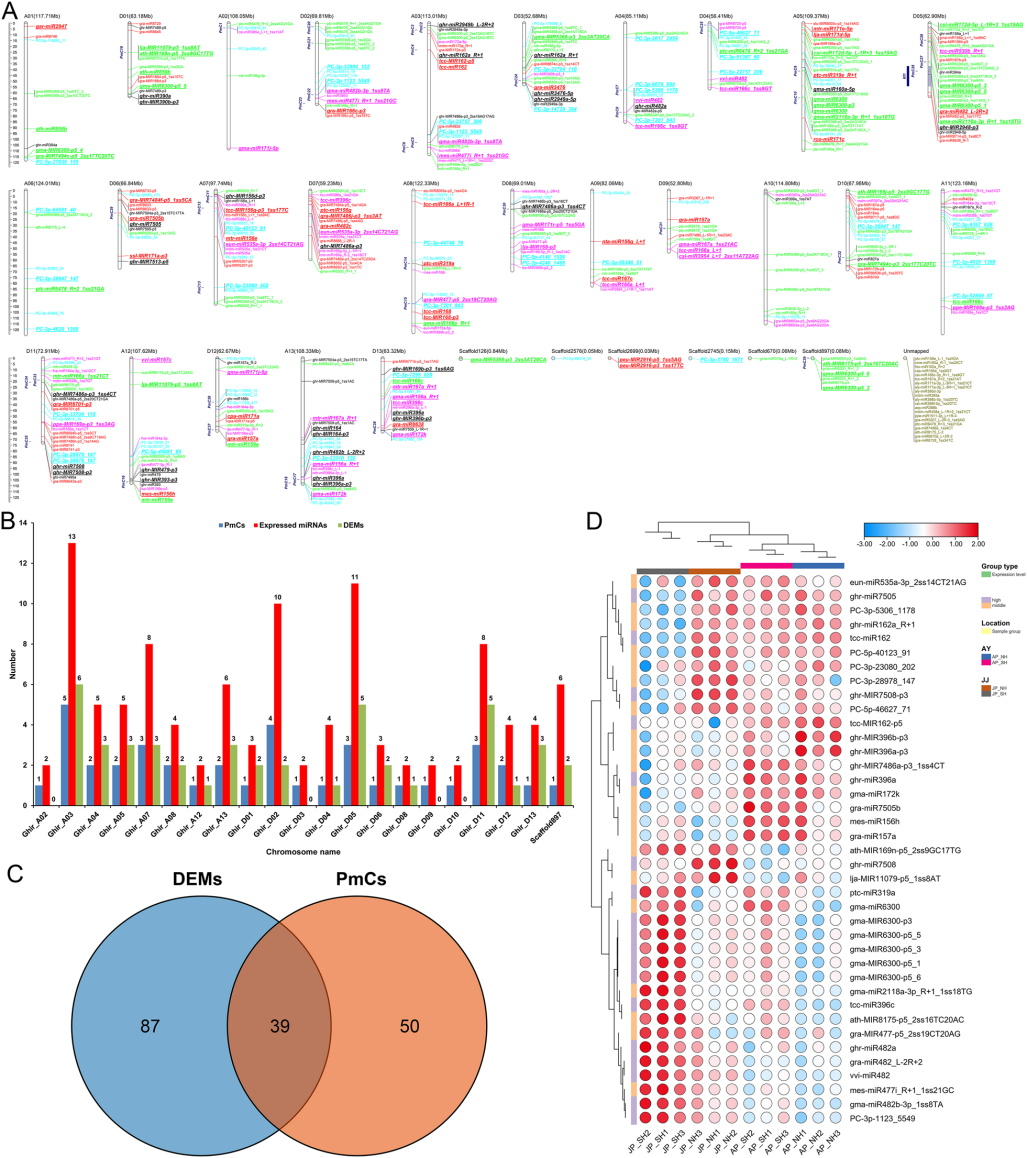
Identification and expression analysis of miRNA clusters. **A **Location distribution of all expressed miRNAs and DEMs contained in Pre-miRNA Clusters (PmCs) on different chromosomes of upland cotton. Different colors indicate different categories of miRNAs, namely black, red, purple, green, olive, and cyan belong to the miRNAs of ‘gp1a’, ‘gp1b’ ‘gp2a’, ‘gp2b’, ‘gp3’ and ‘gp4’ categories, respectively. Among them, miRNAs with bold italics and enlarged fonts and underlined are identified DEMs. The blue columns on the left side of each chromosome or scaffold represent the specific genomic position of all 39 PmCs (Inter-distance < = 50,000 nts), and in particular, the blue column on the left side of Chir_D05 chromosome signifies the mapped interval of the fertility restorer gene *Rf*_*1*_ in our previous study [6]. **B** Statistics of the number of PmCs and their distribution of miRNAs and DEMs on different chromosomes. The X-axis represents chromosome name; the Y-axis and the numbers above each bar represent the PmC and miRNA numbers on each chromosome. **C** Venn diagram showing the number of DEMs in PmCs. **D** Hierarchical cluster analysis of the identified 39 DEMs in PmCs using a heat map drawn by TBtools software. The relative expression levels in the figure are the normalized expression data displayed with log_10_ (norm + 1) value. AP_NH, NH under mild HT stress; AP_SH, SH under mild HT stress; JP_NH, NH under extreme HT stress; JP_SH, SH under extreme HT stress

Some miRNA genes are distributed in clusters on chromosomes and co-transcribed through a promoter and exist in the form of polycistrons [37]. Based on this, we co-localized all 459 known and newly predicted miRNAs on the genome, aiming to find miRNAs that may be simultaneously transcribed in the form of gene clusters (Additional file 2: Table S4). Totally 39 miRNA clusters or Pre-miRNA Clusters (PmCs) containing 106 expressed miRNAs were identified based on 50-kb cluster spacing, including 18 and 20 that were distributed across the eight and 12 chromosomes of At and Dt sub-genomes of upland cotton, and contained 45 and 55 miRNAs, respectively, and one miRNA cluster PmC39 with six miRNAs on Scaffold897 (Fig. 5A, B). Specifically, there were at least two PmCs distributed on ten chromosomes, namely Ghir_A03 (5), Ghir_A04 (2), Ghir_A05 (2), Ghir_A07 (3), Ghir_A08 (2), Ghir_A13 (2), Ghir_D02 (4), Ghir_D05 (3), Ghir_D11 (3) and Ghir_D12 (2), and the remaining chromosomes only carried one PmC. Among them, 45 miRNAs (39 specific) were differentially expressed, including 21 and 22 located in the At and Dt sub-genomes, respectively, and two DEMs on Scaffold897 (Fig. 5C). Heat map analysis of the expression levels of DEMs in the miRNA clusters above found that most highly expressed miRNAs were significantly induced in SH under extreme HT stress compared with NH, especially four MIR482 and six MIR6300 family miRNAs (Fig. 5D). Interestingly, Chir_D05 with restorer gene *Rf*_*1*_ carried three PmCs containing 11 expressed miRNAs, five of which were differentially expressed. It is worth noting that a miRNA cluster, PmC28, was located in the fine-mapped interval of the *Rf*_*1*_ gene, and contained three expressed miRNAs, of which gra-miR482_L-2R + 2 and gma-miR2118a-3p_R + 1_1ss18TG were differentially expressed (Fig. 5), indicating that some HT-responsive miRNAs may be involved in the process of pollen development and fertility restoration in cotton.

### Changes in gene transcript levels are involved in response to HT stress

**Fig. 6:**
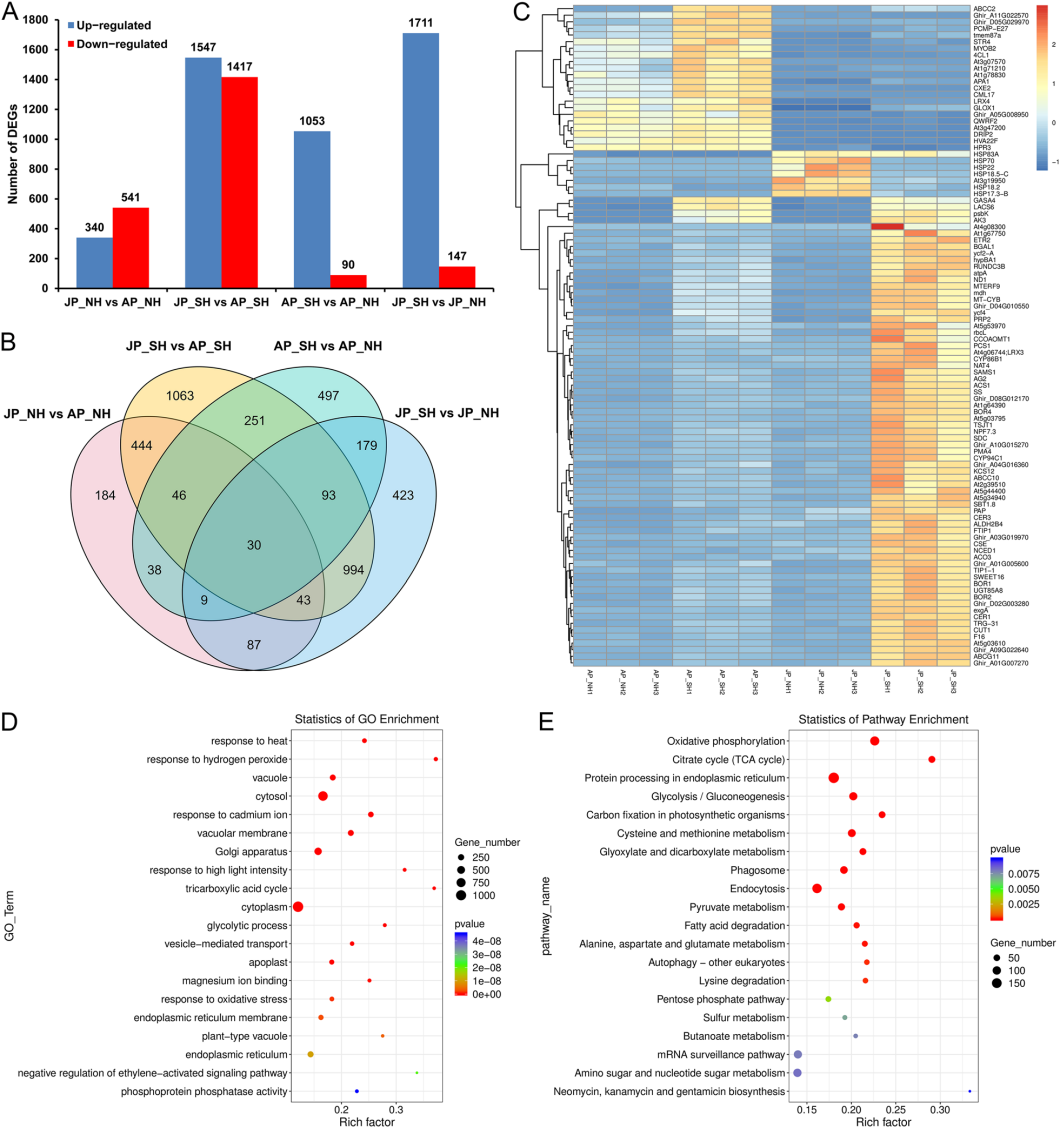
Identification and enrichment analysis of DEGs in NH and SH under mild and extreme HT stress. **A** Number of DEGs that were up- or down-regulated under mild and extreme HT stress. **B** Venn diagram showing the number of unique and shared DEGs. **C** Hierarchical cluster analysis of the top 100 DEGs using a heat map. The abscissa is the sample, and the ordinate is the gene name. The relative expression levels in the figure are the normalized expression data by the *Z*-value method and displayed with log_10_ (FPKM + 1) value; red and dark blue indicate high and low expressed genes, respectively, and the colour bar is on the right of the heat map. **D**, **E** GO (**D**) and KEGG (**E**) enrichment analysis of DEGs. The top 20 GO terms or enriched pathways are plotted based on the significant *P*-value. The size of the circle represents the number of genes, and the color of the circle signifies the* P*-value. The X-axis denotes the enrichment factor, which compares the ratio of genes annotated to a GO term or KEGG pathway among the identified DEGs to the ratio of genes annotated to that GO or pathway among all genes, and the Y-axis indicates the GO or pathway name. AP_NH, NH under mild HT stress; AP_SH, SH under mild HT stress; JP_NH, NH under extreme HT stress; JP_SH, SH under extreme HT stress

To explore whether the changes in target gene expression in response to temperature variation in pollen grains, transcriptome profiles were performed on the same materials used for the sRNA-seq, and a total of 6281 DEGs were identified in the above four comparisons (Additional file 2: Table S13). Among them, 340 and 541 genes were up- and down-regulated, respectively, in JP_NH versus AP_NH, while 1547 up-regulated and 1417 down-regulated genes were identified in JP_SH versus AP_SH. Under mild HT stress, only 1143 genes were differentially expressed (1053 up-regulated and 90 down-regulated genes) for the comparison of AP_SH versus AP_NH; comparatively, a total of 1711 up-regulated and 147 down-regulated were identified between NH and SH under extreme HT stress (Fig. 6A). The Venn diagram showed that the combinations AP_SH versus AP_NH and JP_SH versus JP_NH had only 311 DEMs in common. However, the DEGs only under extreme HT stress (1547) accounted for 57.51% of the total DEMs (2690) of these two combinations, indicating that most DEGs between NH and SH had differential expression changes in response to HT (Fig. 6B). Subsequently, hierarchical clustering analysis of genome-wide differential expression levels for the top 100 DEGs in all 12 samples is shown in Fig. 6C with a heat map. Notably, most genes were up-regulated in SH compared to NH under extreme HT stress, but heat shock protein (HSP)-related genes, such as *HSP70*, *HSP22*, *HSP18.5-C*, *HSP18.2* and *HSP17.3-B* (Fig. 6C), showed significantly reduced expression levels, suggesting that a large number of genes in response to HT may be involved in pollen development.

To gain insight into the potential role of changes in gene transcript levels in cotton pollen development under HT stress, the GO functional enrichment analysis of all 6281 DEGs was performed. Totally 5986 DEGs were annotated to 3571 functional GO categories, of which 682 were significantly enriched (*P*-value < 0.05), including 341 biological processes (BPs), 108 cellular components (CCs), and 233 molecular functions (MFs) (Additional file 1: Fig. S7, Additional file 2: Table S14). This mainly included BPs such as ‘response to heat’, ‘response to hydrogen peroxide’, and ‘response to cadmium ion’. Besides, ‘vacuole’, ‘cytosol’, and ‘vacuolar membrane’ were the three most significant enriched CC terms, while MFs related to ‘magnesium ion binding’, ‘phosphoprotein phosphatase activity’, and ‘UDP-glucuronate decarboxylase activity’ were the three important functional GO categories that were involved (Fig. 6D, Additional file 2: Table S14). To better understand biological functions and regulatory networks, a KEGG pathway enrichment analysis of all 6281 DEGs was also carried out, and only 2553 DEGs were enriched in 137 pathways, of which 28 were significantly enriched (*P*-value < 0.05) (Additional file 2: Table S15). Among these, ‘Oxidative phosphorylation’ was the most significantly enriched pathway, followed by ‘Citrate cycle (TCA cycle)’, ‘Protein processing in endoplasmic reticulum’, and’Glycolysis/Gluconeogenesis’ (Fig. 6E). Additionally, the biosynthesis or metabolism of amino acid and fatty acid pathways, such as ‘Valine, leucine and isoleucine biosynthesis’, ‘Cysteine and methionine metabolism’, ‘Alanine, aspartate and glutamate metabolism’, ‘Arginine and proline metabolism’, ‘Lysine degradation’ and ‘Fatty acid degradation’, were also significantly enriched (Fig. 6E, Additional file 2: Table S15). Based on these results, it is therefore inferred that the homeostasis of energy metabolism-related pathways among carbohydrates, fatty acids and proteins may play a crucial role in pollen development and fertility restoration under HT stress.

### Identification of miRNA targets by degradome sequencing

**Fig. 7:**
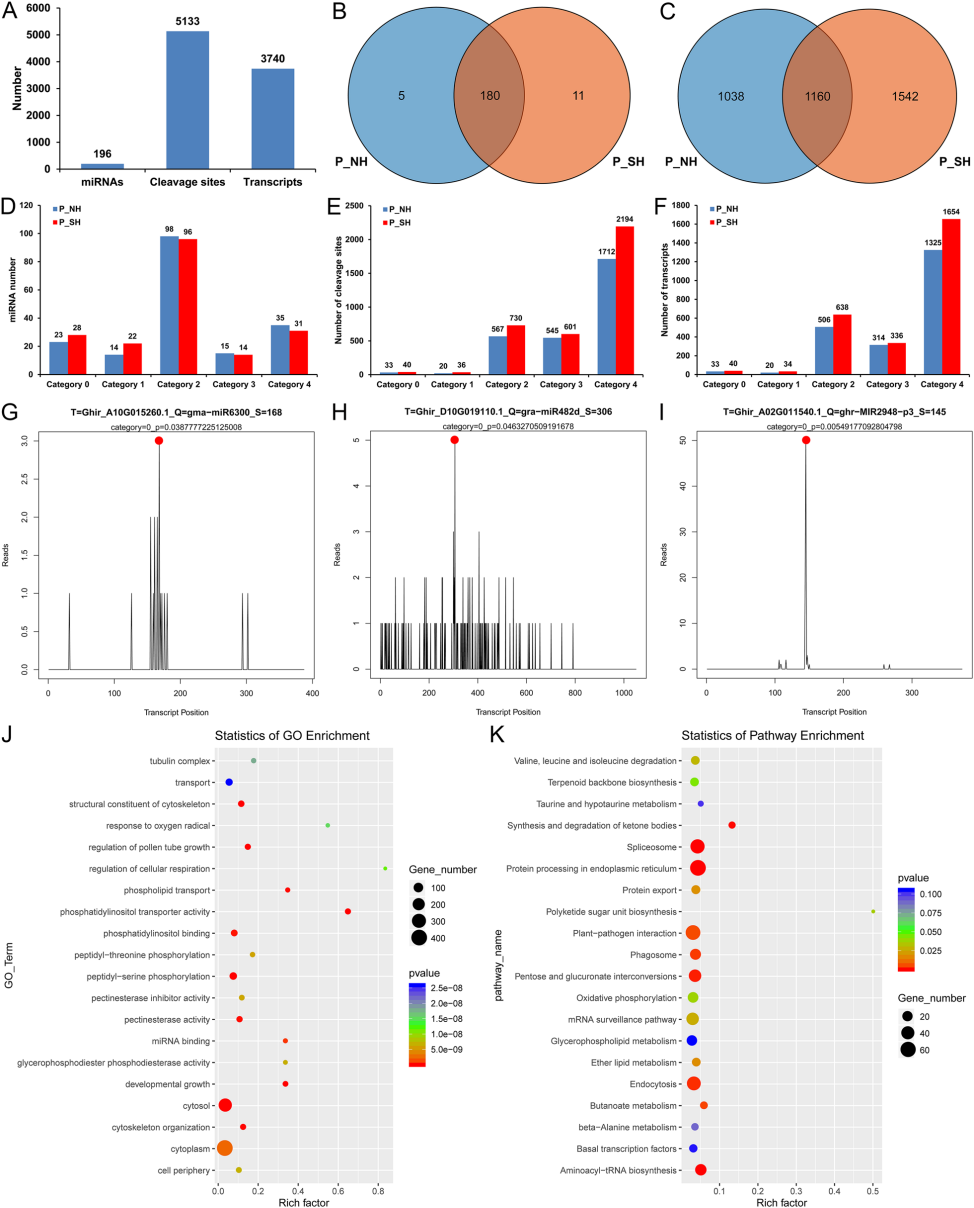
Target identification of miRNAs by degradome sequencing and GO and KEGG enrichment. **A** Number of miRNAs and their target genes. **B**, **C** Venn diagrams showing the number of unique and shared miRNAs **B** and their target genes **C** in P_NH and P_SH. **D**–**F** Categories and statistics of miRNAs (**D**), cleavage sites (**E**), and involving transcripts (**F**). **G** Target plot (T-plot) showing that gma-miR6300 cleaves the *Ghir_A10G015260.1* transcript at the 168th nucleotide position. **H** T-plot showing that gra-miR482d cleaves the *Ghir_D10G019110.1* transcript at the 306th nucleotide position. **I** T-plot showing that ghr-MIR2948-p3 cleaves the *Ghir_A02G011540.1* transcript at the 145th nucleotide position. **J**, **K** GO (**J**) and KEGG (**K**) enrichment analysis of the miRNA targets. P_NH, NH under HT stress; P_SH, SH under HT stress

To determine the target genes of identified heat-responsive miRNAs in pollen grains of cotton CMS-D2 restorer, two degradome sequencing libraries (P_NH and P_SH) for the same pollen samples were constructed to validate the cleavage sites of miRNAs, and more than 15 million raw reads per library were obtained, namely 15,368,314 and 18,907,383 for P_NH and P_SH, respectively. After removing the 3' adaptor and the number of sequences with shorter splicing site tags (< 15-nt), the ratio of unique mappable reads and transcript mapped reads were all larger than 99% and 70%, respectively. Also, the number of covered transcripts ranged from 79,971 to 91,566, which accounted for approximately 55.78% and 63.87% of the total number of input transcripts for P_NH and P_SH, respectively (Table S16), indicating that the degradome sequencing produced a relatively high coverage of degradation fragments. In total, 196 miRNAs and 5,133 cleavage events involving 3,740 candidate target genes were identified (Fig. 7A, Additional file 2: Table S17), of which 180 miRNAs and 1160 target genes were shared between P_NH and P_SH (Fig. 7B, C). Based on the signature number and abundance at each occupied transcript position, these cleaved transcripts were categorized into five categories: 0, 1, 2, 3 and 4. Obviously, category 2 had the most miRNAs, while category 4 exhibited the most cleavage events and transcripts in these two degradome libraries (Fig. 7D–F). Specifically, category 0 contained 23 and 28 miRNAs corresponding to 33 and 40 cleavage events and transcripts in P_NH and P_SH, respectively, and we used the target plot (T-plot) to display the detailed information of three representative miRNAs cleaving the corresponding target genes (Fig. 7G–I). Besides, the other four categories 1, 2, 3 and 4 were counted, and one miRNA and its cleaved target gene were also selected for T-plot in each category (Fig. 7D–F, Additional file 1: Fig. S8).

Based on GO functional enrichment analysis, the targets of the miRNAs described above were significantly enriched in ‘regulation of pollen tube growth’, ‘pollen tube growth’, ‘pollen wall assembly’, ‘pollen germination’, ‘pollen sperm cell differentiation’, and ‘pollen development’ (Fig. 7J, Additional file 2: Table S18). Moreover, KEGG enrichment analysis revealed that 724 of all target genes were enriched into 104 different pathways, of which 16 were significantly enriched (*P*-value < 0.05), including ‘Protein processing in endoplasmic reticulum’, ‘Spliceosome’, ‘Valine, leucine and isoleucine degradation’, ‘Oxidative phosphorylation’, and ‘Terpenoid backbone biosynthesis’ (Fig. 7K, Additional file 2: Table S19). These results suggest that some miRNAs did act in concert with each other to regulate the expression of genes involved in protein metabolism and mitochondrial energy metabolism by cleaving target mRNAs during pollen development and fertility restoration under HT stress.

### Comprehensive analysis of miRNA expression profiles and target genes in response to HT in cotton restorer lines

**Fig. 8:**
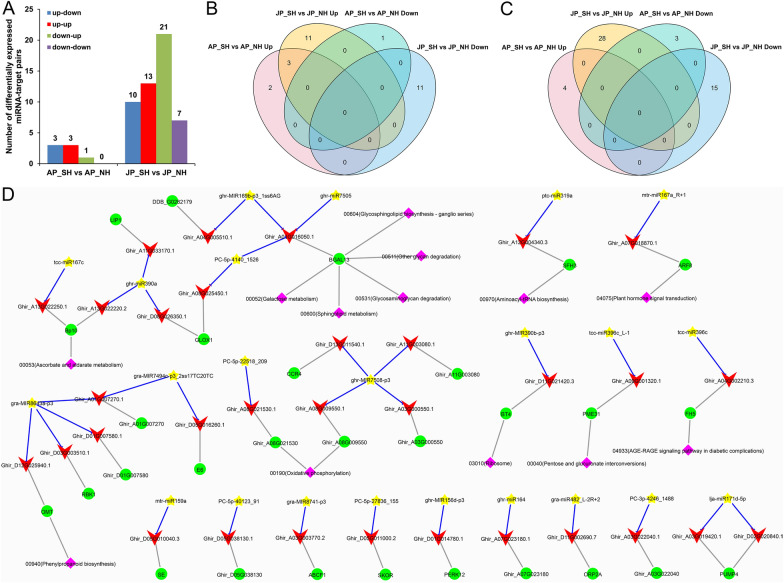
The regulatory miRNA–mRNA interaction pairs in response to HT stress. **A** Number of four representative regulatory types of miRNA–mRNA interaction pairs. **B**, **C** Venn diagrams showing the number of unique and shared miRNAs (**B**) and their target transcripts (**C**) in SH compared with NH under HT. **D** Representative miRNA–mRNA–gene-KEGG regulatory network. Undirected gray lines represent relational pairs, and blue blunt-ended lines represent the inhibitory effect of the miRNAs on the corresponding targets. Yellow star, miRNAs; red vee, target transcripts; green circle, target genes; and purple diamond, KEGG pathways. AP_NH, NH under mild HT stress; AP_SH, SH under mild HT stress; JP_NH, NH under extreme HT stress; JP_SH, SH under extreme HT stress

To further determine the regulatory role of miRNAs during pollen development in response to HT, we integrated combined sRNA, transcriptome, and transcriptome sequencing data to compressively analyze the expression profiles of miRNAs and their corresponding target genes. In total, seven and 51 differently expressed miRNA–target mRNA pairs were identified between SH and NH under mild and extreme HT stress, respectively (Fig. 8A). However, only three up-regulated miRNAs, namely ghr-miR390a, tcc-miR396c, and ptc-miR319a, were shared under either mild or extreme HT stress, but no common target transcripts were found in the two comparative combinations (Fig. 8B, C). Given the inhibitory effects of miRNAs on potential targets in general [38], three up–down and one down–up miRNA–mRNA interaction pairs were identified under mild HT; correspondingly, ten and 21 miRNA–mRNA pairs were found to exhibit opposite up–down and down–up expression patterns under extreme HT, respectively (Fig. 8A, Table 1). Among these 35 negative regulatory pairs, seven miRNAs were found to correspond to multiple targets respectively, and the remaining 16 miRNA–target mRNA pairs were expressed one-to-one (Table 1). Further KEGG enrichment annotation analysis found that these miRNA targets were significantly enriched in ‘Ascorbate and aldarate metabolism’, ‘Oxidative phosphorylation’, ‘Phenylpropanoid biosynthesis’, ‘Plant hormone signal transduction’, and carbohydrate metabolism such as ‘Galactose metabolism’, ‘Other glycan degradation’, ‘Pentose and glucuronate interconversions’ (Additional file 2: Table S20, Fig. 8D).

**Table 1:** The negative regulatory miRNA–mRNA interaction pairs in SH compared with NH under HT stress

miRNA name	Target transcripts	Gene name	TStart	TStop	TSlice	miRNAs		Target transcripts	
						log2 FC	Regulation	log2 FC	Regulation
ghr-MIR156d-p3	Ghir_D01G014780.1	*PERK12*	1778	1798	1790	− 1.32	Down	5.00	Up
ghr-miR164	Ghir_A07G023180.1	*Ghir_A07G023180*	591	613	603	3.02	Up	− 2.39	Down
ghr-MIR169b-p3_1ss6AG	Ghir_A01G016050.1	*BGAL13*	1181	1203	1191	− 1.69	Down	6.32	Up
	Ghir_A04G005510.1	*DDB_G0282179*	598	619	610	− 1.69	Down	4.58	Up
ghr-miR390a	Ghir_A11G033170.1	*LIP1*	1082	1102	1093	0.95	Up	− 3.55	Down
	Ghir_A13G022220.2	*Bp10*	119	138	130	0.95	Up	− 2.21	Down
	Ghir_D08G026350.1	*GLOX1*	1493	1514	1504	2.89	Up	− 2.74	Down
ghr-MIR390b-p3	Ghir_D11G021420.3	*BT4*	1111	1132	1123	1.00	Up	− 15.19	Down
ghr-miR7505	Ghir_A01G016050.1	*BGAL13*	1857	1878	1869	− 0.62	Down	6.32	Up
ghr-MIR7508-p3	Ghir_A03G000550.1	*Ghir_A03G000550*	1060	1083	1073	− 1.26	Down	5.92	Up
	Ghir_A08G009550.1	*Ghir_A08G009550*	1030	1050	1041	− 1.26	Down	16.02	Up
	Ghir_A11G003080.1	*Ghir_A11G003080*	1155	1177	1168	− 1.26	Down	4.27	Up
	Ghir_D13G011540.1	*CCR4*	803	828	818	− 1.26	Down	4.90	Up
gra-miR482_L-2R + 2	Ghir_D11G002690.7	*ORP2A*	168	190	180	1.01	Up	− 3.25	Down
gra-MIR7494c-p3_2ss17TC20TC	Ghir_A01G007270.1	*Ghir_A01G007270*	537	561	550	− 1.37	Down	4.46	Up
	Ghir_D05G016260.1	*E6*	990	1012	1003	− 1.37	Down	4.84	Up
gra-MIR8643a-p3	Ghir_A01G007270.1	*Ghir_A01G007270*	691	711	702	− 1.63	Down	4.46	Up
	Ghir_D01G007580.1	*Ghir_D01G007580*	678	698	689	− 1.63	Down	3.71	Up
	Ghir_D03G003510.1	*RBK1*	424	441	432	− 1.63	Down	5.37	Up
	Ghir_D12G025940.1	*OMT*	610	631	622	− 1.63	Down	3.01	Up
gra-MIR8741-p3	Ghir_A03G003770.2	*ABCF1*	752	772	763	− 0.61	Down	14.08	Up
lja-miR171d-5p	Ghir_A03G019420.1	*PUMP4*	796	821	812	− 1.81	Down	3.33	Up
	Ghir_D02G020840.1	*PUMP4*	607	632	623	− 1.81	Down	6.17	Up
mtr-miR159a	Ghir_D05G010040.3	*SE*	2843	2862	2853	3.83	Up	− 7.05	Down
mtr-miR167a_R + 1	Ghir_A07G018870.1	*ARF8*	2856	2877	2868	4.41	Up	− 6.67	Down
PC-3p-4246_1488	Ghir_A03G022040.1	*Ghir_A03G022040*	871	894	883	− 0.94	Down	3.18	Up
PC-5p-22518_209	Ghir_A08G021530.1	*Ghir_A08G021530*	2222	2242	2233	− 0.54	Down	5.12	Up
PC-5p-27836_155	Ghir_D05G011000.2	*SKOR*	2235	2255	2246	3.50	Up	− 6.60	Down
PC-5p-40123_91	Ghir_D05G038130.1	*Ghir_D05G038130*	242	265	256	− inf	down	3.46	Up
PC-5p-4140_1526	Ghir_A01G016050.1	*BGAL13*	1216	1236	1227	− 2.17	Down	6.32	Up
	Ghir_A08G025450.1	*GLOX1*	1079	1101	1090	− 2.17	Down	3.63	Up
ptc-miR319a	Ghir_A12G004340.3	*SFH3*	904	923	913	2.33	Up	− 3.59	Down
tcc-miR167c	Ghir_A13G022250.1	*Bp10*	429	449	440	1.21	Up	− 8.57	Down
tcc-miR396c	Ghir_A04G002210.3	*FH5*	2631	2651	2642	3.00	Up	− 3.94	Down
tcc-miR396c_L-1	Ghir_A09G001320.1	*PME31*	593	613	604	inf	Up	− 2.69	Down

TStart, TStop and TSlice represent the start site, stop site and cleavage site of the sequence alignment region between the target mRNA and miRNA, respectively

Among these targets, a down-regulated target of mtr-miR167a_R + 1, *Ghir_A07G018870.1*, is a transcription factor encoding auxin response factor 8-like isoform X1 (*ARF8*) and responsible for the auxin signaling transduction. In contrast, two receptor-like protein kinase (RLK) genes *PERK12* and *RBK1* encoding proline-rich RLK and receptor-like cytosolic serine/threonine-protein kinase, respectively, were up-regulated in HT-sensitive SH, while ghr-MIR156d-p3 and gra-MIR8643a-p3 targeting them were inhibited under HT. In the ‘Ascorbate and aldarate metabolism’, the target *Ghir_A13G022250.1* of tcc-miR167c and the target *Ghir_A13G022220.2* of ghr-miR390a are L-ascorbate oxidase homolog encoding pollen-specific protein (*Bp10*), and two other targets of ghr-miR390a, *Ghir_A11G033170.1* (Receptor-like kinase, *LIP1*) and *Ghir_D08G026350.1* (Aldehyde oxidase, *GLOX1*), were also down-regulated in SH compared with NH under HT stress. Moreover, the target gene *Ghir_A09G001320.1* (Pectinesterase 31-like, *PME31*) of tcc-miR396c_L-1 involved in the ‘Pentose and glucuronate interconversions’ pathway and the target genes *Ghir_A01G016050.1* (Beta-galactosidase 13-like precursor, *BGAL13*) and *Ghir_A04G005510.1* (Putative phosphatidylglycerol/phosphatidylinositol transfer protein, *DDB_G0282179*) of ghr-MIR169b-p3_1ss6AG were also significantly different, indicating that the sugar and lipid metabolism and transport pathways are involved in the regulation of cotton male fertility under HT. Based on the above results, we constructed a comprehensive molecular network of miRNA–mRNA–gene-KEGG involved in the regulation of pollen development in response to HT stress (Fig. 8D).

### Excessive auxin accumulation leads to pollen abortion in CMS-D2 restorer line under HT stress by disrupting the homeostasis of JA metabolism

**Fig. 9:**
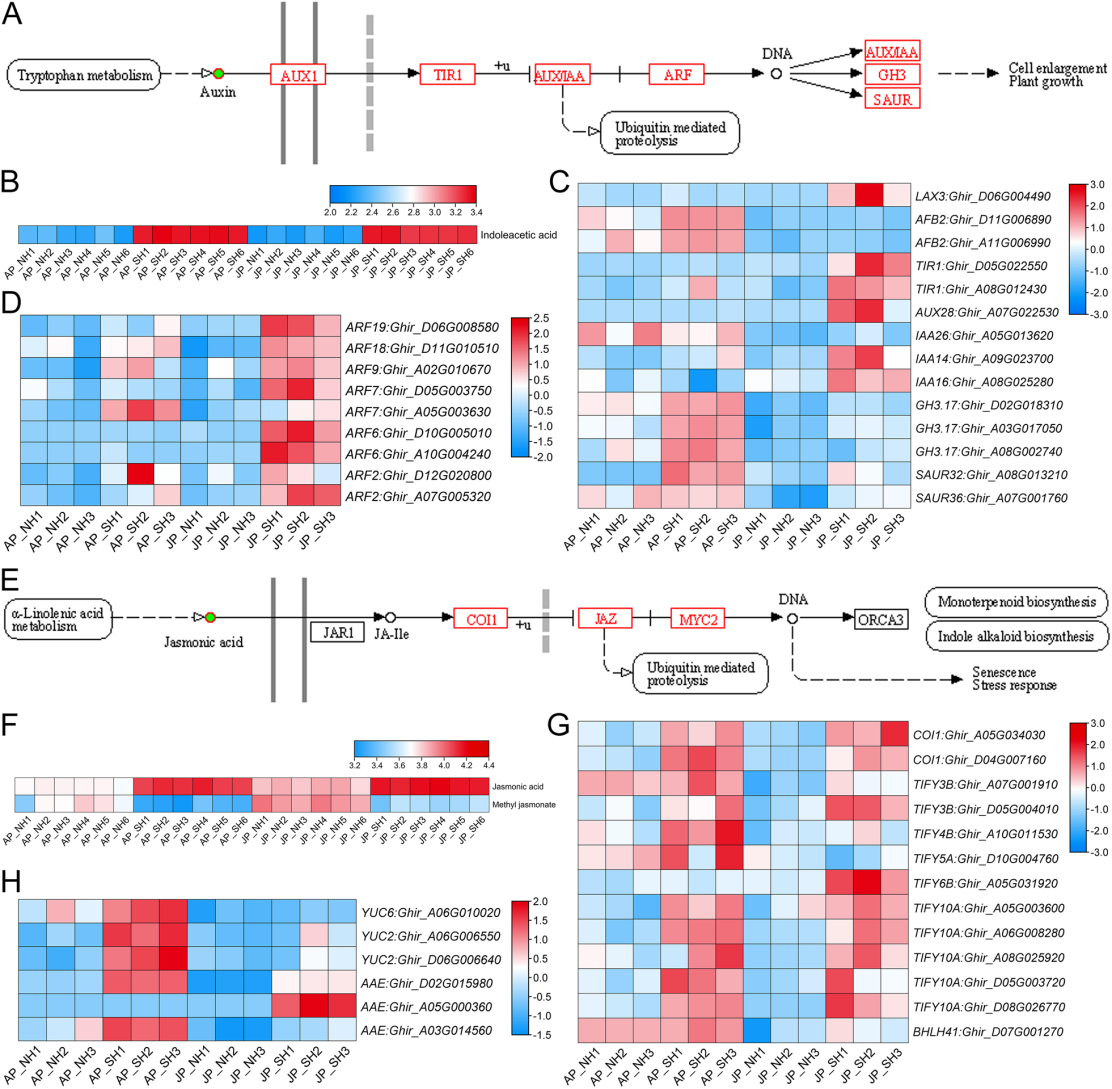
Auxin and JA signal transduction pathways are synergistically involved in the regulation of pollen fertility stability under HT stress. **A** Differentially expressed genes (DEGs) and differential metabolite in the auxin signal transduction pathway. **B** Relative content of indoleacetic acid. (C, D) Heat maps showing expression levels of DEGs involved in auxin signal transduction. **E** DEGs and differential metabolite in the JA signal transduction pathway. **F** Relative contents of JA and methyl jasmonate. **G**, **H** Heat maps showing expression levels of DEGs involved in JA signal transduction and indole alkaloid biosynthesis pathways. Green solid circles and red rectangular boxes indicate differential metabolites and DEGs, respectively. AP_NH, NH under mild HT stress; AP_SH, SH under mild HT stress; JP_NH, NH under extreme HT stress; JP_SH, SH under extreme HT stress

To better demonstrate the involvement of predicted miRNAs and their target genes in pollen fertility stability under HT stress, we also performed metabolite profiling on the same pollen samples [39], and primarily compared the expression levels of related DEGs involved in ‘Plant hormone signal transduction’ pathway (Fig. 9A, E). The relative contents of indoleacetic acid (IAA/auxin) and JA were significantly higher in SH than that in NH, but the active MeJA in SH was obviously down-regulated under HT stress (Fig. 9B, F). Most of the DEGs involved in the auxin signal transduction pathway were significantly up-regulated in SH, among which especially the nine auxin response factor (ARF) genes were more pronounced under extreme HT stress in JJ (Fig. 9C, D), suggesting that heat-activated auxin signal transduction may result in pollen abortion. Similarly, the majority of DEGs involved in the JA signal transduction pathway, especially the two receptor genes *COI1*, were significantly up-regulated in SH under both mild and extreme HT stress (Fig. 9G). Interestingly, DEGs related to the ‘indole alkaloid synthesis’ pathway downstream of JA signal transduction, including *YUC2*, *YUC6* and *AAEs* were also up-regulated in SH (Fig. 9H). In summary, we thus preliminarily infer that HT may disturb the dynamic equilibrium of JA and MeJA synthesis, and that induced JA signaling may activate the expression of downstream auxin synthesis-related genes and cause excessive auxin accumulation, followed by a cascade of auxin signal transduction that finally lead to pollen abortion in HT-sensitive restorer line SH under heat stress (Fig. 1).

## Discussion

### Extensive existing small RNAs are associated with pollen fertility stability of CMS-D2 restorer line under heat stress

The pollen fertility of the restorer line with sterile cytoplasm was significantly reduced under the external continuous HT stress [8], especially in the JJ ecological spot with higher temperature in summer (Fig. 1). Recently, many epigenetic modifications, including small RNAs and DNA methylation, have been reported to be involved in the regulation of plant reproductive development under HT stress [8, 20, 29, 31, 40]. MiRNAs are a class of widely distributed endogenous sRNAs typically ranging from 21 to 24 nt in length and can regulate gene expression post-transcriptionally by guiding the degradation of their mRNA targets or inhibiting translation [24, 25], which act an important role in regulating plant anther development in response to HT stress [20, 29]. Here, sRNAs of 21–24 nt in length were found to account for more than 66% of all reads in each library, and all samples showed the highest richness at 24-nt (Fig. 2D, E). This was basically consistent with the distribution of sRNA abundance during anther development in previous studies in cotton [38, 41] and other plants, such as rice [27], soybean [42], and tomato [43], whereas there were exceptions for sRNAs with the highest abundance at 21-nt in wheat [44] and another recent study in soybean [29]. Given RdDM is the major epigenetic pathway that can guide de novo CHH methylation on strand-specific DNA sequences via a combination of 24-nt small-interfering RNAs (siRNAs) [35]. In *Arabidopsis*, siRNAs derived from retrotransposons accumulate in pollen and sperm cells, signifying that epigenetic reprogramming occurs during reproductive development [45]. Thus, the changes in the ratio of 24/21 nt sRNAs may reflect alterations in DNA methylation levels during anther development. Contrary to the trend in response to HT in soybean [29], we found that the ratio of 24/21 sRNAs decreased under extreme HT stress in both HT-tolerant restorer NH and HT-sensitive SH, and the magnitude of decline was more obvious in NH (Fig. 2F). Moreover, the proportion of VsRNA reads in SH was significantly less than that of NH, especially in the JJ spot with extreme HT (Fig. 2B, C). These results suggest that potential epigenetic changes do occur during pollen development, and widespread DNA demethylation in response to HT is essential for maintaining normal anther development in cotton [8].

It is becoming a consensus that miRNA sequences had base-bias characteristics, and the first base of miRNAs with different lengths showed different preferences [27, 41]. In this study, 19–22 nt miRNAs in mature pollens frequently started with ‘U’ as the first base (Fig. 3D, Additional file 2: Table S10), which is in accordance with the recent studies in anthers of rice photoperiod- and thermo-sensitive genic male sterile (PTGMS) line [27] and cotton [41]. By contrast, the first base of 24-nt miRNAs was mainly ‘C’ with a proportion of 45.16% in cotton pollen but ‘A’ in rice and cotton anthers (45.80% and 83.10%, respectively) [27, 41]. In addition, the 5'start and 3'end bases of mature miRNAs were mainly 'U' and 'C', respectively (Figs. 3E, Additional file 1: Fig. S4E, F, Additional file 2: Table S11). Taken together, such findings indicated that the sequence preference of mature miRNAs is indeed not only universal but also varies among different plants, regardless of different tissue selection and/or experimental treatments.

### Heat-responsive miRNAs and their clusters participate in regulating the pollen development and fertility restoration in cotton

Totally 104 shared miRNAs were identified in all samples, and specific miRNAs only accounted for only a small portion (Fig. 3C); besides, significantly more of whether up- or down-regulated DEMs were found under extreme HT than that under mild HT (Fig. 4A). These findings indicate that the effect of miRNAs on pollen fertility in response to HT may be mainly by regulating the expression abundance (up- or down-regulation) of existing miRNAs in plant cells, rather than directly inducing the expression of more novel miRNAs [31, 46]. By integrating multi-omics data, we constructed a comprehensive network of miRNA–mRNA–gene-KEGG involved in regulating pollen fertility stability in response to HT (Fig. 8D). Previous research in *Arabidopsis* revealed that miR156 enhances the HT stress memory by regulating its targets of *SQUAMOSA promoter-binding protein-like* (*SPL*) transcription factor genes [33]. In soybean, all members of the gma-miR156 family were repressed by HT in both HT-tolerant and HT-sensitive CMS-based F_1_ combinations, and overexpression of gma-miR156b in *Arabidopsis* led to pollen sterility under HT stress [29]. Consistent with recent studies in PTGMS rice [27] and cotton [20], we also found ghr-MIR156d-p3 was suppressed under HT stress but its target *PERK12* encoding proline-rich RLK was up-regulated in HT-sensitive SH; gra-MIR8643a-p3 and its target gene *RBK1* encoding receptor-like cytosolic serine/threonine-protein kinase were also found to have similar expression patterns in response to HT (Fig. 8, Table 1). Typical RLKs are a large family of membrane proteins sensing extracellular signals to regulate plant growth and development, and stress responses. In the model plant *Arabidopsis thaliana* and other plant species, RLK-mediated signaling pathways play crucial roles in regulating the reproductive process and stress response by sensing different ligand signals [47]. In rice, two leucine-rich repeat RLKs, Thermo-Sensitive Genic Male Sterile 10 (TMS10) and its close homolog TMS10-LIKE (TMS10L) redundantly control tapetal degeneration and microspore viability, and the pollen fertility in *tms10* mutant is sensitive to HT [48]. Thus, miR156 and miR8643 may play important roles in cotton pollen fertility in response to HT by regulating their corresponding targets *PERK12* and *RBK1*, but more experiments are needed to dissect the connections in detail. Growing evidence has supported that ROS-dependent cellular metabolic processes are involved in anther development [7, 9, 49, 50], and unbalanced ROS metabolism in cotton anthers cause male sterility under HT stress [8, 40]. Non-enzymatic ROS scavenging systems, including glutathione and ascorbate, function in the removal of hydroxyl radicals and singlet oxygen [51], and the target *Bp10* of tcc-miR167c and ghr-miR390a are involved in the ‘Ascorbate and aldarate metabolism’ pathway, were also significantly down-regulated in SH under HT (Fig. 8, Table 1). This indicates that heat-induced miR167 and miR390 may disrupt the oxygen homeostasis in plant cells at the key stage of anther development by inhibiting the expression of related genes in ROS scavenging system, thus leading to pollen abortion in cotton [50]. Besides, the target gene *PME31* of tcc-miR396c_L-1 involved in the ‘Pentose and glucuronate interconversions’ pathway, and the target genes *BGAL13* and *DDB_G0282179* of ghr-MIR169b-p3_1ss6AG also showed negative correlation with the corresponding miRNAs (Fig. 8, Table 1), signifying that HT-responsive miRNAs mediating the sugar and lipid metabolism and transport pathways are also involved in regulating cotton male fertility [17, 19, 39].

Some miRNA genes are distributed in clusters on chromosomes and co-transcribed through an identical promoter and exist in the form of polycistrons [37], and are related to the CMS occurrence in cotton [41]. Here, we identified 39 miRNA clusters containing 106 expressed miRNAs in cotton pollen grains, and the number of PmCs and their miRNAs also including DEMs did not differ greatly between At and Dt sub-genomes (Fig. 5A–C). Notably, miRNA cluster PmC28 was located within the fine-mapped interval of the fertility restorer gene *Rf*_*1*_ [6, 52], and contained two DEMs, gra-miR482_L-2R + 2 and gma-miR2118a-3p_R + 1_1ss18TG were significantly up-regulated in SH under extreme HT (Fig. 5A, D). MiR482/2118, as one of the conserved miRNA superfamilies originating from gymnosperms, has evolved with diverse functions in core-angiosperms. It predominantly regulates *NUCLEOTIDE BINDING SITE-LEUCINE-RICH REPEAT* (*NBS-LRR*) genes in eudicots, which are vital for plant disease resistance; comparatively, it mainly targets numerous long non-coding RNAs (lncRNAs) in monocot grasses, functioning as an essential component in plant reproduction [53]. Presently, some studies have shown that miR2118 and miR2275 trigger the production of 21- and 24-nt reproductive phased small-interfering RNAs (phasiRNAs), respectively, and these stage-specific phasiRNAs may play key roles in microgametogenesis in rice [54, 55] and maize [56]. In rice, the PGMS trait of NK58S is controlled by two loci, *Pms1* and *Pms3*, of which *Pms1* encodes a lncRNA *PMS1T* acting as a phasiRNAs-producing locus, and the SNP mutation from G to T in *Pms1* influences miR2118 binding and increases the processing of 21-nt reproductive phasiRNAs, thus down-regulating unknown target genes involved in tapetum programmed cell death and finally resulting in the male sterility under long-day conditions [57]. In addition, phasiRNAs triggered by miR482 also participate in abiotic stress resistance, such as responding to drought stress in poplar [58]. Thus, we deduce here that some heat-responsive miRNAs and their clusters, especially the 21-nt reproductive phasiRNAs triggered by the miR482/2118 superfamily, may be involved in the process of pollen development and fertility restoration in cotton under HT stress.

### Homeostatic modulation of both auxin and JA signaling in response to HT is essential for normal pollen development in cotton

Endogenous phytohormones auxin and JA homeostasis and their subsequent signal transduction pathways have been proven to be involved in the response to HT stress during anther development [15–20]. Auxin was confirmed essentially for floral development in an earlier study, as *yuc1yuc4*, *yuc2yuc6*, all of the triple and quadruple mutants of the four auxin biosynthesis-related *YUC* genes (*YUC1*, *YUC2*, *YUC4*, and *YUC6*) always lost male fertility [59]. In cotton, it has been verified that exogenous IAA treatment results in pollen abortion and anther indehiscence under long-term HT stress [19, 20, 40, 60], and overexpression of miR160 can cause the activation of auxin response through suppressing the transcription levels of its targets *ARF10* and *ARF17*, and ultimately lead to anther indehiscence [20]. Consistently, we also found the relative content of IAA in SH was obviously higher than in NH, and most of the DEGs involved in the auxin signaling were significantly up-regulated in SH under extreme HT in JJ spot (Fig. 9A–D). However, endogenous auxin levels of anthers in barley and model plant *Arabidopsis thaliana* significantly decreased under HT stress, and exogenous auxin application could completely rescue male sterility triggered by HT [18]. Similarly, suppressed auxin signaling via overexpressing miR157 in cotton also leads to more sensitivity to HT stress, producing indehiscent anthers and sterile pollen grains [20]. Recent research in strawberry flowers found that all CeO_2_ NP treatments significantly increased pollen grain numbers, and promoted the pollen grain germination rates and pollen tube elongations resulting from the increased IAA and cytosolic Ca^2+^ contents [61]. These findings suggest that too high or too low levels of auxin are both detrimental to male fertility in flowering plants. Taken together, we infer that a finely regulated auxin biosynthesis and its signal transduction pathway must be necessary for anther development and fertility restoration in cotton under HT stress.

Previous studies in *Arabidopsis* have shown that anther dehiscence occurs earlier in auxin-perception mutants than wild-type due to enhanced JA biosynthesis [15], and the JA signal was also proven earlier to be essential for anther dehiscence [62]. Here, the relative content of JA were significantly higher in SH than in NH, whereas the content of active MeJA in SH was obviously down-regulated under HT stress (Fig. 9F), indicating that excessive auxin accumulation in HT-sensitive CMS-D2 restorer SH activates its signal transduction, which may lead to anther indehiscence by disrupting the homeostasis of JA metabolism. This is basically consistent with the results of our previous research [16] and other reports on cotton [17, 63]. In *Arabidopsis*, *ARF6* and *ARF8* have been experimentally confirmed to be required for activation of *DAD1* (*DEFECTIVE IN ANTHER DEHISCENCE1*) expression and therefore to regulate JA biosynthesis; loss of *ARF6* and *ARF8* genes was found to disrupt JA production and henceforth cause delayed or indehiscence, and petal elongation and reduced filament [22], which can also be reverted by exogenous JA application [23]. Moreover, *Arabidopsis* miR167 can regulate both female and male reproduction by controlling the expression patterns of *ARF6* and *ARF8* responsible for auxin signal transduction [64]. Here, it was found that as a target for up-regulating mtr-miR167a_R + 1, *ARF8* was significantly down-regulated in heat-sensitive SH under HT stress (Fig. 8D, Table 1). Additionally, most of the DEGs involved in the JA signaling and the DEGs related to the ‘indole alkaloid synthesis’ pathway downstream of JA signal transduction, including *YUC2*, *YUC6,* and *AAEs* were all significantly up-regulated in SH under HT (Fig. 9E–H). These results suggest that auxin biosynthesis and signal transduction might be concurrently regulated by miR167 and JA signal. Therefore, we conclude that the expression of miR167 induced by HT disrupts the dynamic balance of JA and MeJA production by inhibiting the transcription level of *ARF8*, and the JA signaling induced by certain unknown ways may activate the expression of downstream auxin synthesis-related genes and causing excessive auxin accumulation, followed by a cascade of auxin signal transduction that ultimately result in the emergence of negative effects of CMS-D2 cytoplasm, namely anther indehiscence and pollen abortion in SH under HT. On the other hand, *ARF8* may directly be a negative regulatory factor in auxin response, similar to the inhibition of *ARF10* and *ARF17* by miR160 in cotton, which activates auxin signaling and finally results in anther indehiscence and male sterility under HT stress [20]. However, the detailed molecular mechanisms of how the heat-responsive miRNA167 and its target *ARF8* fine-regulate the coordination of these two plant hormones in the stability of pollen fertility restoration for CMS-D2 cotton still need to be further explored.

### Fertility restoration stability regulated by various miRNAs and their targets under HT stress

**Fig. 10:**
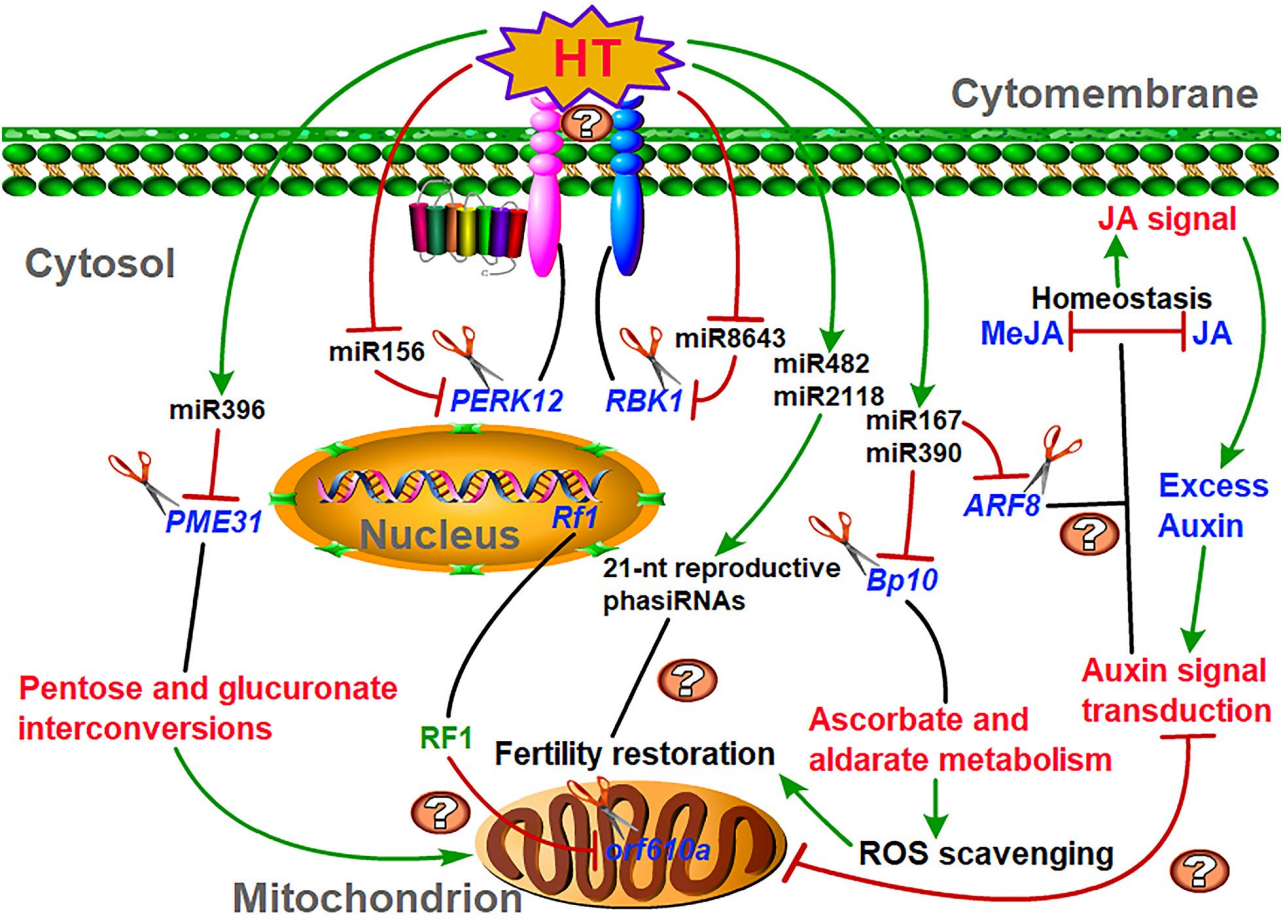
A proposed model showing heat-responsive miRNAs involved in regulating the stability of pollen fertility restoration for CMS-D2 cotton under HT stress. The lines with arrows and blunt ends in the figure signify the promotion and inhibition modes respectively, and the accompanying question marks represent unknown action modes or connections

Although our research group has recently successfully identified for the first time the sterile gene *orf610a* located in the mitochondrial genome and controlling CMS-D2 in cotton sterile line, and preliminarily explained the reasons for its pollen abortion [65], the detailed regulatory mechanisms of how the nuclear *Rf*_*1*_ gene acts on *orf610a* to finally achieve pollen fertility restoration and the potential roles of miRNAs in fertility stability under HT remain indistinct. Based on our results of this study and those of previous studies, we proposed a potential model for various heat-responsive miRNAs to regulate the stability of pollen fertility restoration under HT stress (Fig. 10). In HT-sensitive restorer line SH, the expression levels of miR156 and miR8643 were inhibited by HT, which as a result, two membrane receptor-like protein kinase genes *PERK12* and *RBK1* were up-regulated, and their sensing extracellular HT signals might be excessively amplified through unknown ways, leading to the destruction of pollen fertility stability. HT-induced miR167 and miR390 may disturb the oxygen homeostasis by inhibiting the expression of related genes in ROS scavenging system; meanwhile, the over activated auxin signal and the inhibited sugar metabolism may be caused by HT via promoting the expression of miR167 and miR396, respectively, and further affecting the transcriptional levels of their corresponding targets or interfering with the JA metabolic homeostasis, which ultimately leads to anther indehiscence and pollen abortion under HT stress by unknown ways. Besides, some heat-responsive miRNAs and their clusters, especially the 21-nt reproductive phasiRNAs triggered by the miR482/2118 superfamily, may be also involved in the process of fertility restoration in cotton. However, how these heat-responsive miRNAs precisely regulate their targets, and whether the signal pathways involved have synergistic or antagonistic effects on cotton male fertility stability under HT stress still need to be further investigated.

## Conclusions

In summary, the present study performed an integrated small RNA, transcriptome, degradome, and hormone analysis and revealed the importance of heat-responsive miRNAs in regulating male fertility stability of the CMS-D2 restorer line. We identified differentially expressed miRNAs, miRNA clusters and their targets, and further, constructed a comprehensive molecular network of miRNA–mRNA–gene-KEGG comprising 35 pairs of miRNA/target genes involved in the regulation of pollen development under HT stress. Furthermore, combining metabolomic data suggested that auxin and JA synthesis and their signal transduction pathways in response to HT may play crucial co-regulatory roles during anther development in cotton. Collectively, our results will contribute to better understanding the regulatory mechanisms of the negative effects of sterile cytoplasm on pollen development under heat stress.

## Materials and methods

### Plant materials and growth conditions

Two upland cotton restorer lines with different cytoplasm, whose pollen fertility stability showed obvious differences in performance under continuous HT stress in field were employed in this study, including the HT-tolerant restorer line NH with normal upland cotton cytoplasm, and its isonuclear alloplasmic near-isogenic line (NIL) SH (formerly also named ZBR) with CMS-D2 cytoplasm, which is sensitive to HT [8]. All cotton materials were developed in detail as described in our previous studies [6, 36, 39], and seeds were harvested and conserved at the Cotton Heterosis Utilization Laboratory (our research group), the Institute of Cotton Research of Chinese Academy of Agricultural Sciences (ICR-CAAS).

NH and SH were sown at the end of April 2020 at Baibi East Experimental Farm, ICR-CAAS, Anyang (AY), Henan Province, China (36°10′N, 114°35′E), which is located in the cotton area of the Yellow River basin, and the experimental field of the Cotton Research Institute of Jiang Xi Province, Jiujiang (JJ), Jiangxi Province, China (29°71′N, 115°85′E), which is located in the cotton area of Yangtze River basin; cotton field management practices followed local recommendations. At the end of July and early August, the average temperatures for at least five consecutive days in AY reached 35 °C in the daytime and 27 °C at night, which are defined as continuous mild HT stress; similarly, in JJ, the average temperatures for at least five consecutive days were approximately 38 °C in the daytime and 30.5 °C at night, which are defined as continuous extreme HT stress. Mature pollens were collected centrally and combined from 30 representative plants for each genotype in each replication under mild and extreme HT conditions, respectively, and snap-frozen in liquid nitrogen and then stored at − 80 °C in a freezer until use. Based on different ambient temperature conditions, four samples used in this study were designated as follows: AP_NH, NH under mild HT stress; AP_SH, SH under mild HT stress; JP_NH, NH under extreme HT stress; JP_SH, SH under extreme HT stress.

### Phenotype analysis and pollen vigor determination

Flower morphology of NH and SH under mild and extreme HT stress was observed and recorded intensively daily, respectively, and images of representative flowers or anthers were photographed using a Canon EOS 80D digital camera (https://www.canon.com.cn/product/80d/). To determine pollen vitality under HT, mature pollen grains from the typical flower phenotypes of NH and SH were stained with Benzidine-α-Naphthol [66], and images were captured under a bright field using an Olympus SZX16 research stereo microscope system (https://lifescience.evidentscientific.com.cn/en/microscopes/stereo/szx16/).

### Small RNA sequencing library construction and bioinformatics analysis

#### RNA extraction and small RNA library construction

Totally 12 samples in this study were used for small RNA (sRNA) sequencing that contained two cotton NILs, NH and SH; two temperature conditions, mild and extreme HT; and three biological replicates. Total RNA was extracted from the pollens of each sample using the TIANGEN RNAprep Pure Plant Plus Kit (Polysaccharides & Polyphenolics-rich; DP441) following the vendor’s protocol. To ensure the quality for library construction, the total RNA amount and purity were quantified using a NanoDrop ND-1000 (NanoDrop, Wilmington, DE, USA), and RNA integrity for each sample was also assessed by an Agilent 2100 Bioanalyzer system with RIN number > 8.0. About 1 µg of total RNA was used to prepare and construct the sRNA library using a TruSeq Small RNA Sample Prep Kit (Illumina, San Diego, CA, USA), and the detailed process was described in our recent research [41]. After cluster generation, the libraries were sequenced on an Illumina HiSeq 2500 platform (LC Bio, Hangzhou, China) according to the manufacturer’s recommended protocol, and 50-bp single-end sequencing reads were generated.

#### Sequencing data processing and identification of candidate miRNAs and their clusters

Raw data were subjected to an in-house program, ACGT101-miR (LC Sciences, Houston, Texas, USA) to obtain the clean reads by removing the low-quality reads with 5’ adapter contaminants or without the 3’ adapter tag and inserted segment, reads containing poly-N > 10%, reads containing continuous poly A, T, G or C, and other common sRNA families (rRNA, tRNA, snRNA, snoRNA) and repeats. All sRNA tags were aligned to the upland cotton TM-1 genome using Bowtie 2 [67] to analyze their expression and distribution information on the cotton reference genome [68]. Then, unique sequences with length in 18 ~ 25 nucleotides (nt) were mapped to specific cotton precursors in miRBase 22.0 database by the BLAST search tool to identify known miRNAs and novel 5p- and 3p-derived miRNAs. Length variations at both 3’ and 5’ ends and one mismatch inside of the sequence were permitted in the alignment. The unique sequences mapping to specific cotton mature miRNAs in hairpin arms were considered to be known miRNAs, whereas mapping to the other arm of known specific cotton precursor hairpin opposite to the annotated mature miRNA-containing arm was identified as novel 3p- or 5p-derived miRNA candidates. The remaining sequences were mapped to other selected species precursors (with the exclusion of specific cotton) in miRBase 22.0 database by BLAST search, and the mapped pre-miRNAs were further BLASTed against the specific cotton genome to obtain their genomic locations. The above two we both defined as known miRNAs. Besides, the unmapped sequences were BLASTed against the specific cotton genome, and the hairpin RNA structures containing sequences were predicated from the flank 120-nt sequences by RNAfold software (http://rna.tbi.univie.ac.at/cgi-bin/RNAWebSuite/RNAfold.cgi). Specifically, the criteria for secondary structure prediction of single-stranded RNA sequences were described in detail as follows: (1) number of nt in one bulge in stem (≤ 12); (2) number of base pairs in the stem region of the predicted hairpin (≥ 16); (3) length of the hairpin (up and down stems + terminal loop ≥ 50); (4) length of the hairpin loop (≤ 200); (5) cutoff of minimum free energy (MFE, kCal/mol ≤ − 15); (6) number of nt in one bulge in the mature region (≤ 4); (7) number of biased bulges in the mature region (≤ 2); (8) number of biased errors in one bulge in the mature region (≤ 2); (9) number of base pairs in the mature region of the predicted hairpin (≥ 12); (10) number of errors in the mature region (≤ 4); (11) percent of mature in stem (≥ 80).

In our analysis pipeline, the known miRNAs used the miFam database (http://www.mirbase.org/ftp.shtml) to search for families, and novel miRNA precursors were submitted to the Rfam database (http://rfam.xfam.org) to search for and browse Rfam families. Moreover, the base bias or preference on the first position of identified mature miRNAs with a certain length and on each position of all mature miRNAs was also counted. According to the physical location of miRNA precursors in the cotton genome, miRNA clusters based on 50-kb cluster spacing were also identified referring to our recent research [41], and MapChart software [69] was used to perform the chromosome co-location analysis to show their distribution.

#### Analysis of differential expressed miRNAs and target gene prediction

Custom scripts were used to obtain the miRNA counts, and the frequency for each miRNA in 12 libraries was standardized to the expression level of transcripts per million (TPM) using the following formula: normalized TPM = (actual miRNA counts/total counts of clean tags) *10^6^. MiRNA expression fold changes between samples were calculated using log_2_(TPM of sample 1/TPM of sample 2), of which three biological replicates were merged into the mean value. Differential expression of miRNAs between or among different samples based on normalized TPM values was analyzed by selectively using the Student* t*-test or *ANOVA* according to the specific experiment design, and the significance threshold here was set to be corrected* P*-value < 0.05 in each test. Also, the genes targeted by the most abundant miRNAs were predicted by PsRobot [70], and computational target prediction algorithms TargetFinder [71] were used to identify miRNA binding sites.

### RNA sequencing and data analysis

Transcriptome sequencing was conducted with three biological replicates on the same materials used for sRNA sequencing analysis (LC Bio, Hangzhou, China). After filtering the low-quality reads containing adapters and poly-N from the raw data using the Trimmomatic software [72], clean reads were obtained and then mapped to the TM-1 reference genome using TopHat [73]. Cuffdiff software was then used to calculate the FPKM (fragments per kilobase of exon per million fragments mapped) value of each gene and determine differential expression between or among different samples based on a negative binomial distribution model [74], and transcripts with a corrected *P*-value < 0.05 were considered differentially expressed genes (DEGs) [75]. The GOseq R package [76] was used for Gene Ontology (GO) functional categories analysis, and the KOBAS software [77] was used to test the statistical enrichment of the DEGs in the Kyoto Encyclopedia of Genes and Genomes (KEGG) pathways, among which GO terms or KEGG pathways with an adjusted *P*-value < 0.05 were considered to be significantly enriched.

### Degradome sequencing and target identification

Two degradome libraries, P_NH and P_SH (NH and SH mixed under both mild and extreme HT stress, respectively), were prepared by mixing an equal amount of total RNA from six pollen samples into 30 ug without considering the difference in ecological spots and constructed referring to the methods described in previous studies [78–80]. In brief, the detailed procedures for the construction of the degradome sequencing library were described in our recent study [41] and another research in PTGMS rice [27]. The average insert size for the final cDNA libraries was 200 ~ 400 bp, and then 50-bp single-end sequencing was performed on an Illumina HiSeq 2500 platform (LC Bio, Hangzhou, China).

After base calling, the raw data were first pre-processed using a Perl script to remove the reads with adaptors, that had an unknown base poly-N or low-quality reads to obtain clean tags. The extracted sequencing reads were subsequently used to identify potentially cleaved targets of miRNAs and other small RNAs through the Cleveland pipeline [81]. Clean tags of the degradome were aligned to the GenBank and Rfam 11.0 databases to obtain annotation information for the rRNA, tRNA, snRNA and snoRNA, and were also mapped to the TM-1 genome to gain cDNA sense and antisense tags [68]. Only the perfect matching alignments for the given reads mapped to cDNA or mRNA sequences were kept for further degradation analysis. All resulting reads (t-signatures) were reverse-complemented and then aligned to the miRNAs identified in this study, while no more than four alignment scores were allowable. Alignments where the degradome sequence position corresponding to the tenth or eleventh nucleotides of specific miRNA were retained and scored. The targets were selected and categorized as 0, 1, 2, 3 or 4, specifically defined as previously described [27]. Additionally, to easily analyze the RNA degradation patterns, T-plots were illustrated based on the distribution of signatures and abundances along these transcripts to show the cleavage sites of miRNAs. Finally, all the identified targets were subjected to BLASTX analysis to search for protein similarity and then to GO [76] and KEGG [77] enrichment analysis to construct the miRNA–gene-GO/KEGG regulatory network.

### Determination and comparative analysis of metabolite content

Metabolic profiling analysis with six biological replicates was also conducted on the same pollen samples, of which the relative contents of indoleacetic acid, jasmonic acid (JA) and active methyl jasmonate (MeJA) both in NH and SH under HT stress were determined and compared. The detailed procedures of metabolite extraction and mass spectrometry analysis were described in our recent study [39].

### Statistical analysis and graphical presentation

Each diagram in this study signifies the results of multiple independent experiments (n ≥ 3), and the values are presented as means ± standard deviation (SD). The statistical significance analyses between NH and SH under HT stress were estimated using a two-tailed unpaired Student’s *t*-test, and a *P*-value < 0.05 was considered significantly different. UpSet Venn and Directed Network diagrams in this manuscript were generated using OmicShare online tools (https://www.omicshare.com/tools/home/soft/getsoft.html). Also, an integrative toolkit TBtools [82] was used to graphically display some results of this study, such as a bending heat map and Venn diagrams.

### Supplementary Information

**Additional file 1: Fig. S1.** Evaluation of small RNA sequencing (sRNA-Seq) data quality. (a) Pearson correlation analysis between samples in the sequencing libraries. (b) Three-dimensional principle component analysis (PCA) factorial map showing the largest components of variance. PCA analysis was performed using the “vegan” package of R software based on normalized read counts of all miRNAs obtained from sRNA-Seq. **Fig. S2.** Classification statistics of repetitive sequences in total and unique data of 12 sRNA-seq libraries. **Fig. S3.** Rfam sequence category in total and unique data of 12 sRNA-seq libraries. **Fig. S4.** Statistics of different types of miRNAs identified in each sample and their base preference analysis. (A, B) Number of pre-miRNAs (A) and unique miRNAs (B) in different categories in each sample. (C, D) The first base preference of mature miRNAs in ‘gp1_3’ (C) and ‘gp4’ (D) categories. (E, F) Base preference in different positions of mature miRNAs in ‘gp1_3’ (E) and ‘gp4’ (F) categories. **Fig. S5.** Analysis of miRNA family and conservation. (A) Family analysis of identified miRNAs. (B) Statistics on the frequency of the identified miRNAs in other species. **Fig. S6.** Number of all expressed miRNAs and DEMs on different chromosomes of upland cotton. The X-axis represents different chromosomes; the Y-axis and the numbers above each bar represent the miRNA numbers on each chromosome. **Fig. S7.** GO functional classification of all DEGs. The X-axis indicates the GO categories, and the Y-axis indicates the number of DEGs. **Fig. S8.** T-plots of miRNAs in categories 1–4 verified by degradome sequencing. (A) T-plot showing that gra-miR157a cleaves the *Ghir_A05G033800.1* transcript at the 101st nucleotide position. (B) T-plot showing that PC-5p-4140_1526 cleaves the *Ghir_D01G002960.1* transcript at the 707th nucleotide position. (C) T-plot showing that gra-MIR477-p5_2ss19CT20AG cleaves the *MSTRG.26509.1* transcript at the 178th nucleotide position. (D) T-plot showing that ghr-miR7505 cleaves the *Ghir_D05G035640.1* transcript at the 770th nucleotide position.

**Additional file 2: Table S1.** Overview of reads from raw data to cleaned sequences in 12 small RNA sequencing (sRNA-seq) libraries. **Table S2.** Classification statistics of repetitive sequences in total and unique data of 12 sRNA-seq libraries. **Table S3.** Length distribution of counts of total and unique sRNA sequences in 12 sRNA-seq libraries. **Table S4.** Detailed summary of identified known and predicted miRNAs and their expression levels in this study. **Table S5.** Statistics of the number of pre-miRNAs and unique miRNAs in different categories for each sample. **Table S6.** Length distribution of all identified miRNAs. **Table S7.** MiFam-based family analysis of identified miRNAs. **Table S8.** Conservation of the identified miRNAs with other species. **Table S9.** Conservation profile of the identified miRNA family members. **Table S10.** The first base preference of mature miRNAs. **Table S11.** Base preference in different positions of mature miRNAs. **Table S12.** Identification and expression level analysis of differentially expressed miRNAs. **Table S13.** Identification and expression level analysis of differentially expressed genes. **Table S14.** GO functional enrichment analysis of all DEGs. **Table S15.** KEGG pathway enrichment analysis of all DEGs. **Table S16.** Overview of degradome sequencing data output for P_NH and P_SH. **Table S17.** Target gene and predicted cleavage site information validated through degradome sequencing analysis in P_NH and P_SH. **Table S18.** GO enrichment analysis of the miRNA targets. **Table S19.** KEGG enrichment analysis of the miRNA targets. **Table S20.** KEGG enrichment annotation analysis of miRNA targets with negative regulatory relationship under HT stress.

## Data Availability

The datasets used and/or analyzed during the current study are available from the corresponding authors on reasonable request.
